# A novel bZIP protein, Gsb1, is required for oxidative stress response, mating, and virulence in the human pathogen *Cryptococcus neoformans*

**DOI:** 10.1038/s41598-017-04290-8

**Published:** 2017-06-22

**Authors:** Seon Ah Cheon, Eun Jung Thak, Yong-Sun Bahn, Hyun Ah Kang

**Affiliations:** 1 Department of Life Science, Chung-Ang University, Seoul, 06974 Korea; 2 Center for Fungal Pathogenesis, Seoul National University, Seoul, 08826 Korea; 3 Department of Biotechnology, Center for Fungal Pathogenesis, Yonsei University, Seoul, 03722 Korea

## Abstract

The human pathogen *Cryptococcus neoformans*, which causes life-threatening meningoencephalitis in immunocompromised individuals, normally faces diverse stresses in the human host. Here, we report that a novel, basic, leucine-zipper (bZIP) protein, designated Gsb1 (general stress-related bZIP protein 1), is required for its normal growth and diverse stress responses. *C. neoformans gsb1*Δ mutants grew slowly even under non-stressed conditions and showed increased sensitivity to high or low temperatures. The hypersensitivity of *gsb1*Δ to oxidative and nitrosative stresses was reversed by addition of a ROS scavenger. RNA-Seq analysis during normal growth revealed increased expression of a number of genes involved in mitochondrial respiration and cell cycle, but decreased expression of several genes involved in the mating-pheromone-responsive MAPK signaling pathway. Accordingly, *gsb1*Δ showed defective mating and abnormal cell-cycle progression. Reflecting these pleiotropic phenotypes, *gsb1*Δ exhibited attenuated virulence in a murine model of cryptococcosis. Moreover, RNA-Seq analysis under oxidative stress revealed that several genes involved in ROS defense, cell-wall remodeling, and protein glycosylation were highly induced in the wild-type strain but not in *gsb1*Δ. Gsb1 localized exclusively in the nucleus in response to oxidative stress. In conclusion, Gsb1 is a key transcription factor modulating growth, stress responses, differentiation, and virulence in *C. neoforman*s.

## Introduction

Infection of the central nervous system with a basidiomycete fungal pathogen, *Cryptococcus neoformans*, can lead to cryptococcal meningitis (CM), killing hundreds of thousands of people annually, especially immunocompromised patients. Nearly 1 million cases of HIV/AIDS-associated CM occur annually worldwide, causing nearly 625,000 deaths within three months of diagnosis; the majority of CM cases occur in sub-Saharan Africa^1^. Although the introduction of highly active antiretroviral therapy (HAART) has decreased the death rate in HIV/AIDS patients with cryptococcal infection in developed countries, CM is still a major problem in third-world countries with high HIV prevalence^1–3^. *C. neoformans* is found in nature, e.g., in soil, trees, and bird guano. During the progression of infection, *C. neoformans* encounters a number of stressors, such as temperature fluctuations, oxidative and osmotic stresses, nutrient limitation, physiological pH, and high CO_2_ levels in the human host^4^. Therefore, the ability to sense, respond, and adapt to host stressors is crucial for survival and proliferation of this opportunistic pathogen^4^.

*C. neoformans* has evolutionarily conserved and distinct signaling pathways to counteract various environmental/host stressors. These pathways include high-osmolarity glycerol response, Ras/cAMP/protein kinase A (PKA), Rim101, Ca^2+^/calcineurin, target of rapamycin (TOR), protein kinase C (PKC), and the unfolded protein response (UPR)^5–13^. External stressors can activate specific signaling pathways through specific receptors or sensors that then relay their signals by engaging some other downstream signaling molecules. This leads to the activation and expression of a group of target genes via one or many specific transcription factors (TFs). Among TF families, the basic, leucine-zipper (bZIP) family is one of the largest and the most conserved family of proteins that play crucial physiological roles in various eukaryotes. The bZIP domain generally contains 60–80 amino acids, and a basic region for DNA binding and nuclear translocation, followed by a leucine-zipper motif for dimerization^14^. Several stress-associated *C. neoformans* TFs, such as Yap1, Yap4, Atf1, HapX, and Hxl1, were previously classified as bZIP proteins^13, 15–17^. A recent systematic, functional analysis of *C. neoformans* TFs revealed that among 178 analyzed putative TFs, 12.7% harbor the bZIP domain^18^. The cryptococcal bZIP TFs characterized so far are reportedly involved in cellular responses to oxidative, osmotic, and endoplasmic reticulum (ER) stresses as well as several biological functions, including iron uptake or storage, sexual development, high-temperature growth, drug resistance, and virulence. More recently, a unique bZIP TF, named Bdr1, was shown to regulate resistance to gamma radiation in *C. neoformans* by controlling expression of the DNA-damage response genes^19^.

In this study, we constructed a set of *C. neoformans* null-mutant strains lacking the putative bZIP proteins, which were previously shown *in silico* to harbor the bZIP domain similar to that of Hac1/Xbp1 homologs^13^, the bZIP transcriptional factors controlling the UPR pathways in eukaryotes. We then assessed the growth phenotype of the mutant strains under several stress conditions. We report that a novel bZIP protein, designated general stress-related bZIP protein 1 (Gsb1), is involved in growth, sexual differentiation, and responses to diverse stresses, particularly against oxidative stresses, and is required for potent virulence of *C. neoformans*. Based on the transcriptome profiling and growth phenotypes, such as mitochondrial dysfunction and abnormal cell division, of the *gsb1*Δ mutant strain, we propose that Gsb1 is a major regulator required for responses to oxidative stress and during other developmental processes, including mating and cell cycle.

## Results

### A novel *C. neoformans* bZIP protein, Gsb1, is involved in diverse stress responses

In a previous study on UPR mediated by the Hxl1 bZIP TF in *C. neoformans* ^13^, we identified four other open-reading frames (ORFs) (CNAG_00871, CNAG_03976, CNAG_07560, and CNAG_07940) as putative Hac1/Xbp1 homologs, but with low similarity in the bZIP domain. These proteins have an N-terminal bZIP domain (Supplementary Fig. S1a), except for CNAG_03976, and they all show slightly different expression levels under ER stress conditions^13^. To investigate whether CNAG_00871, CNAG_03976, CNAG_07560, and CNAG_07940 play any roles in the *C. neoformans* UPR, we disrupted every ORF by a *NAT*-resistance marker on the background of the *C. neoformans* H99 serotype A, and obtained some deletion mutants for CNAG_00871, CNAG_07560, and CNAG_07940, but not CNAG_03976. We confirmed the correct deletion of the targeted bZIP genes by using Southern blotting (Supplementary Fig. S2). Then, we analyzed two independent mutants of each gene for their sensitivity to ER stressors and heat stress, which are the two major UPR-dependent phenotypic traits (Supplementary Fig. S1b). Unlike the UPR mutants (*ire1*Δ and *hxl1*Δ)^13^, the deletion mutants of CNAG_00871, CNAG_07560, and CNAG_07940 did not show any increased sensitivity to tunicamycin and DTT compared to the wild-type (WT) strain, indicating that the three bZIP proteins were not involved in the ER stress responses. Notably, however, the CNAG_07560 deletion mutant showed apparently slow growth even under a non-stressed condition (YPD medium at 30 °C) and increased sensitivity to high (39 °C) or low (16 °C) temperature (Supplementary Fig. S1b).

To further explore physiological roles of the bZIP proteins, we investigated growth phenotypes of the bZIP mutants on solid media containing various stress-causing reagents, including cell-wall stressors—Congo red (CR), SDS, and caffeine; oxidative stressors—diamide and H_2_O_2_; and osmotic stressors—KCl and NaCl (Supplementary Fig. S1c). While the deletion mutants of CNAG_00871 and CNAG_07940 displayed growth phenotypes almost identical to that of the WT strain, the CNAG_07560 deletion mutant showed high sensitivity to oxidative, osmotic, and cell-wall stressors. These results indicated that the bZIP protein encoded by CNAG_07560 is required for both normal growth and stress responses in *C. neoformans*. Therefore, CNAG_07560 was named a general stress bZIP protein 1 (Gsb1) in *C. neoformans*. Notably, compared to the *cac1*Δ, *cna1*Δ, *cpk1*Δ, *hog1*Δ, and *mpk1*Δ mutant strains, which reportedly are highly sensitive to oxidative stress (Supplementary Fig. S1c), the *gsb1*Δ mutant showed the highest sensitivity to oxidative stress.

### A scavenger of reactive oxygen species reverses the hypersensitivity of *gsb1*Δ to oxidative stress

**Figure 1:**
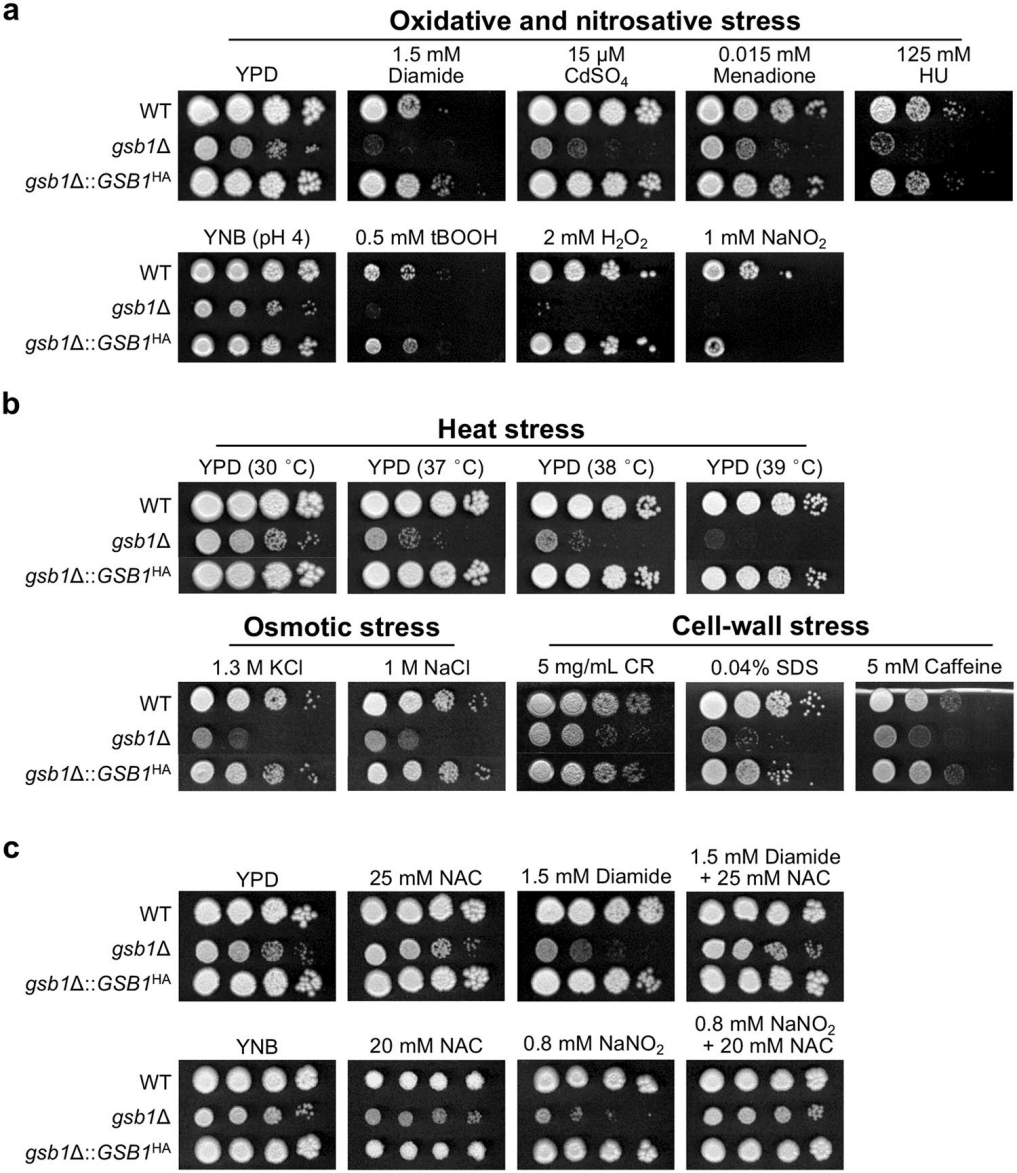
Growth phenotype of the *C. neoformans gsb1*Δ mutant strain under various stress conditions. (**a**) Spotting analysis of *C. neoformans gsb1*Δ strain in the presence of various oxidative stressors. Hydroxyurea (HU); *Tert*-butyl-hydrogen peroxide (tBOOH). (**b**) Spotting analysis of *C. neoformans gsb1*Δ strain under heat stress (37, 38, 39 °C), osmotic stress (KCl, NaCl) and cell-wall stress (Congo Red:CR, SDS, caffeine) conditions. (**c**) Effect of a ROS scavenger, N-acetyl-L-cysteine (NAC), on the growth of *gsb1*Δ strain under oxidative stress conditions. Wild-type H99 strain (WT); *gsb1*-null mutant strain (*gsb1*Δ); the *gsb1*Δ strains complemented with expression of the C-terminal HA tagged Gsb1 (*gsb1*Δ*::GSB1* ^HA^). For spotting analysis, *C. neoformans* cells, grown in YPD overnight, were washed in distillated water twice, diluted serially, and spotted on YPD or YNB media containing the reagents at the indicated concentrations, and then incubated for 4 days at 30 °C.

We further investigated the hypersensitivity of the *gsb1*Δ mutant to oxidative stress by assessing its growth in the presence of additional oxidative stressors—CdSO_4_, menadione, and *tert*-butyl-hydrogen peroxide, and nitrosative stressors—NaNO_2_ and hydroxyurea. The *gsb1*Δ mutant exhibited hypersensitivity to all the tested oxidative and nitrosative stressors (Fig. 1a), indicating that Gsb1 plays a general role in anti-oxidative and anti-nitrosative stress responses. The growth defects of the *gsb1*Δ mutant were fully restored by reintroduction into its native locus of the WT allele of *GSB1* gene, encoding Gsb1 tagged with human influenza hemagglutinin (HA) at its C-terminus, Gsb1^HA^ (Fig. 1). Expression of Gsb1^HA^ was confirmed by western blotting using the anti-HA antibody (Supplementary Fig. S3). Interestingly, although Gsb1 is composed of 403 amino acids with an expected size of 46 kDa, Gsb1^HA^ was detected at an apparent molecular weight of ~70 kDa. Gsb1 is predicted to have 21 putative phosphorylation sites by *in silico* analysis using NetPhosYeast 1.0^20^, but the phosphatase treatment experiment suggested that the increased molecular weight of Gsb1 was not attributed to phosphorylation (Supplementary Fig. S3). The sensitivity of the *gsb1*Δ mutant to heat, osmotic, and cell-wall stressors was also reversed by expression of Gsb1^HA^ (Fig. 1b), verifying its pleiotropic roles.

Next, we tested the possibility that Gsb1’s loss of function may cause elevation of intracellular reactive oxygen species (ROS) levels, leading to such hypersensitivity to oxidative stressors. Indeed, addition of the ROS scavenger N-acetyl-L-cysteine restored the normal growth of the *gsb1*Δ mutant under oxidative and nitrosative stress conditions (Fig. 1c). These results strongly indicated that Gsb1 might contribute to the detoxification of ROS or reactive nitrogen species and protect *C. neoformans* against such stressors.

### Gsb1 is associated with mitochondrial function

**Figure 2:**
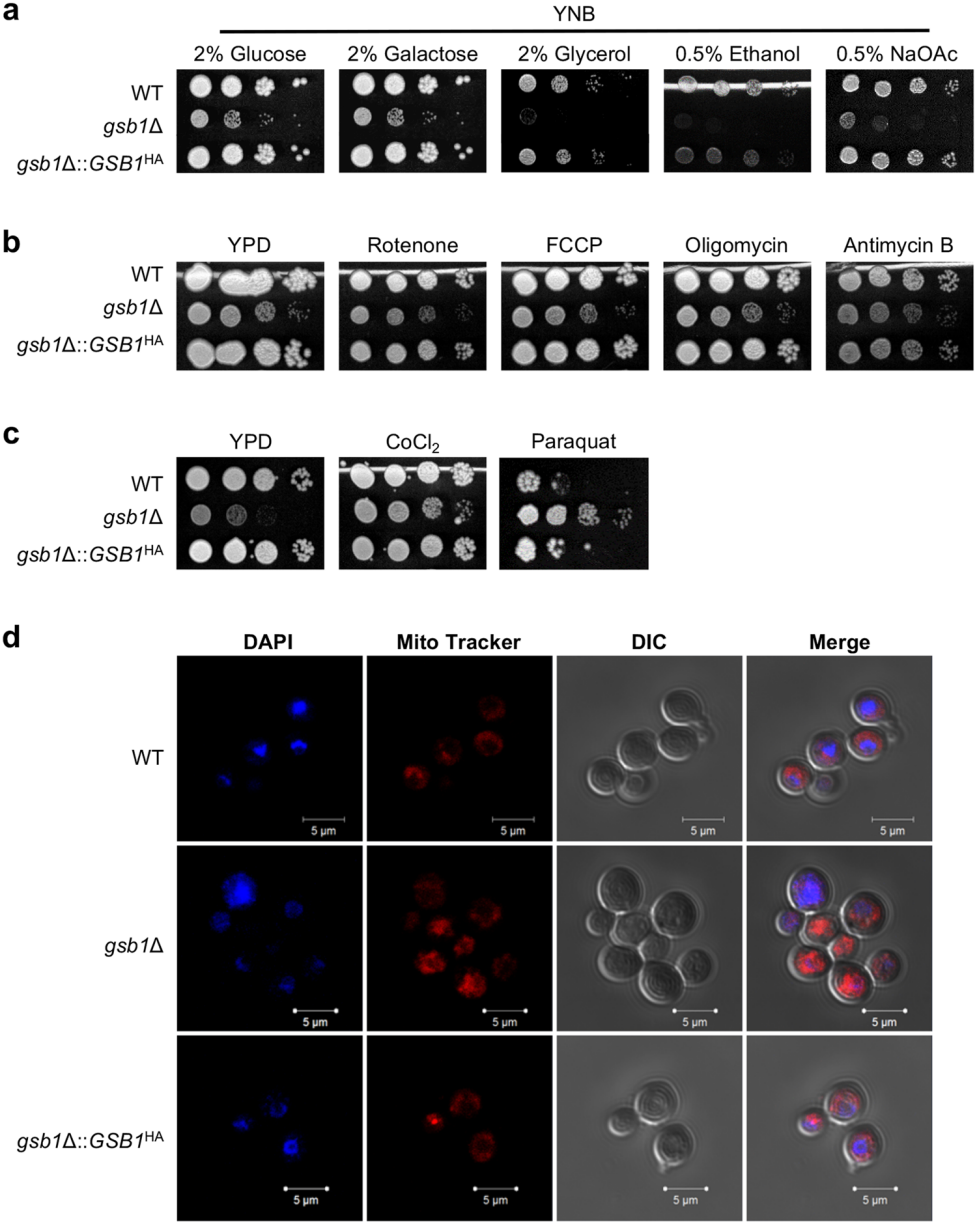
Analysis of mitochondrial function and integrity in the *gsb1*Δ strain. (**a**) Growth analysis on non-fermentable carbon sources. Strains were spotted on the synthetic minimal media containing glucose (2%), galactose (2%), glycerol (2%), ethanol (0.5%), or sodium acetate (0.5%) as carbon source. (**b**) Growth analysis of *gsb1*Δ in the presence of inhibitors of mitochondrial ETC. Yeast cells were spotted on YPD plates containing the indicated ETC inhibitors, such as rotenone (1 mg/mL), FCCP (0.5 μg/mL), oligomycin (10 μg/mL), and antimycin B (1 μg/mL). (**c**) Growth analysis of *gsb1*Δ in the presence of hypoxia-mimicking or ROS-generating reagents. Yeast cells were spotted on YPD plate containing CoCl_2_ (0.7 mM) or paraquat (0.5 mM). All plates were incubated at 28 °C for 4 days. (**d**) Staining analysis of mitochondria. *C. neoformans* cells were grown to early log phase and subjected to confocal microscopy. Nuclei were stained with DAPI and mitochondria were labeled with MitoTracker Red CMXRos.

Mitochondria are a major source of ROS, which directly target the mitochondrial lipids and the complexes of the electron transport chain (ETC)^21^. *S. cerevisiae* mitochondrial mutant strains, either having defective respiration or overproducing ROS, cannot grow on non-fermentative carbon sources, which mitochondria-dependent respiration essentially uses for cellular growth^22, 23^. Interestingly, more severe growth defects were observed in the *gsb1*Δ mutant grown on non-fermentative carbon sources, such as glycerol, ethanol, and sodium acetate (Fig. 2a), indicating that Gsb1 might be involved in mitochondrial function required for growth on such carbon sources. We thus examined whether the growth of the *gsb1*Δ mutant could be influenced by inhibitors of mitochondrial ETC (Fig. 2b). Compared to the WT strain, the *gsb1*Δ mutant was almost similarly sensitive to ETC inhibitors, such as antimycin B (Complex III inhibitor), FCCP (uncoupler of electron transport from oxidative phosphorylation), oligomycin (ATP synthase inhibitor), and rotenone (Complex I inhibitor). The majority of cryptococcal mutants with reduced respiration rates were reportedly hypersensitive to hypoxia-mimicking agents, such as cobalt chloride (CoCl_2_), or low-oxygen conditions^24^. No difference in growth was observed between the WT and *gsb1*Δ mutant in the presence of CoCl_2_ in culture, further suggesting that the *gsb1*Δ mutant does not have defective respiration (Fig. 2c). Notably, however, the *gsb1*Δ mutants were found to be resistant to paraquat, a superoxide generator, at a level that induced hypersensitivity in the WT strain (Fig. 2c). *S. cerevisiae* mutants with mitochondrial dysfunction, such as defective respiration or defective mitochondrial carriers, are shown to resist paraquat^25, 26^. Thus, our results suggest that the mitochondrial dysfunction of the *gsb1*Δ mutant partly results from abnormal function of mitochondrial carriers.

By staining with the MitoTracker dye (CMXRos), we assessed whether mitochondrial integrity was compromised in the *gsb1*Δ mutant. Interestingly, the *gsb1*Δ mutant accumulated the MitoTracker dye more than the WT strain, and this over-accumulation was reversed by Gsb1^HA^ expression (Fig. 2d). Because the MitoTracker passively diffuses across the plasma membrane and then accumulates in active mitochondria in a potential-dependent manner^27, 28^, we speculate that the mitochondrial membrane potential in the *gsb1*Δ mutant might be higher than that in the WT strain. Altogether, our results suggest that the dysfunctional mitochondrial metabolism in the *gsb1*Δ mutant might partly result from the combined effect of an over-activated ETC, generating excess ROS, and defective ROS detoxification in the absence of functional Gsb1.

### Comparative transcriptome profiling of WT and *gsb1*Δ strains

**Figure 3:**
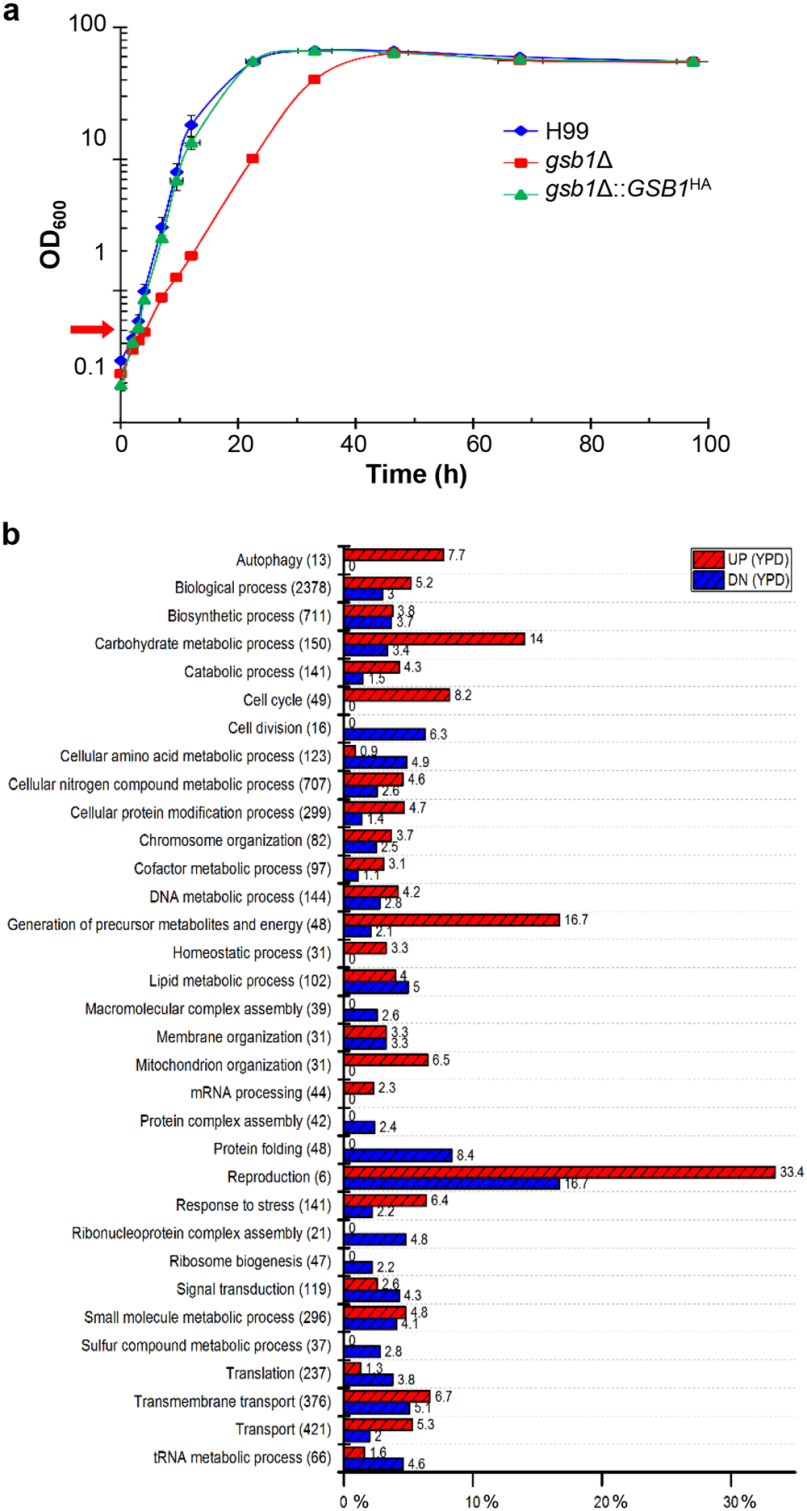
Comparative RNA-Seq analysis of the WT and *gsb1*Δ strains under normal growth condition. (**a**) Time course analysis of *C. neoformans* growth under normal conditions. *C. neoformans* cells were grown in shaker flasks containing complex YPD media at 28 °C for 4 days. (**b**) Gene ontology (GO) enrichment analysis of the differentially expressed genes between WT and *gsb1*Δ strains under normal growth condition. For RNA-Seq analysis, WT H99 and *gsb1*Δ strains were inoculated at OD_600_ = 0.25 in YPD broth and harvested at OD_600_ = 0.5 (early exponential phase). The sampling point was indicated with a red arrow in Fig. 3a. GO enrichment was analyzed using the AgBase^45^ tool, Parenthesis: Total gene number within each functional category, %: percentage of genes showing differential expression.

As indicated by spotting analysis in Supplementary Fig. S1, *GSB1* disruption resulted in retarded cell growth: two-fold slower growth rate of *gsb1*Δ than that of the WT strain was observed during exponential phase even under normal growth conditions on YPD (Fig. 3a). To identify the downstream genes whose expression levels are affected by the absence of Gsb1 under normal growth conditions, we carried out RNA-Seq analysis and compared the transcriptome profile of the WT and the *gsb1*Δ mutant cultivated in YPD up to the early exponential growth phase (Fig. 3b). Under normal conditions, 491 (7.1%) and 274 (4%) genes were upregulated and downregulated, respectively, by more than 2-fold in the *gsb1*Δ mutant compared to that in the WT strain; this indicates that Gsb1 might serve as a transcriptional activator and repressor for different subsets of genes. The gene ontology (GO) enrichment analysis revealed that the expression of some genes involved in autophagy, carbohydrate and nitrogen metabolism, cell cycle, metabolite and energy generation, and mitochondrial organization is significantly increased in the *gsb1*Δ mutant (Supplementary Table S3). Notably, a set of mitochondrial genes associated with ETC, such as genes encoding the cytochrome c oxidase subunits (CO1, CO2, CO3), NADH:ubiquinone oxidoreductases (ND4) and dehydrogenases (ND1, ND2, ND3, ND6), and ATP synthase subunit (ATP6), were upregulated in the *gsb1*Δ mutant (Supplementary Table S3), which was validated by qRT-PCR analysis (Fig. S4). These findings agree well with the finding that the *gsb1*Δ mutant was more actively stained with the MitoTracker dye (Fig. 2d). Moreover, expression of genes involved in stress responses, autophagy, and protein modification were higher in the *gsb1*Δ mutant than in the WT strain, indicating that the *gsb1*Δ mutant is chronically under stress (Supplementary Table S3). In contrast, a few other functional genes, which are significantly repressed in the *gsb1*Δ mutant under normal growth conditions, are involved in cell division, macromolecular and ribonucleoprotein complex assembly, ribosome biogenesis, protein folding, translation, and tRNA metabolism. This explains why the *gsb1*Δ mutant exhibits a low growth rate under normal conditions.

**Figure 4:**
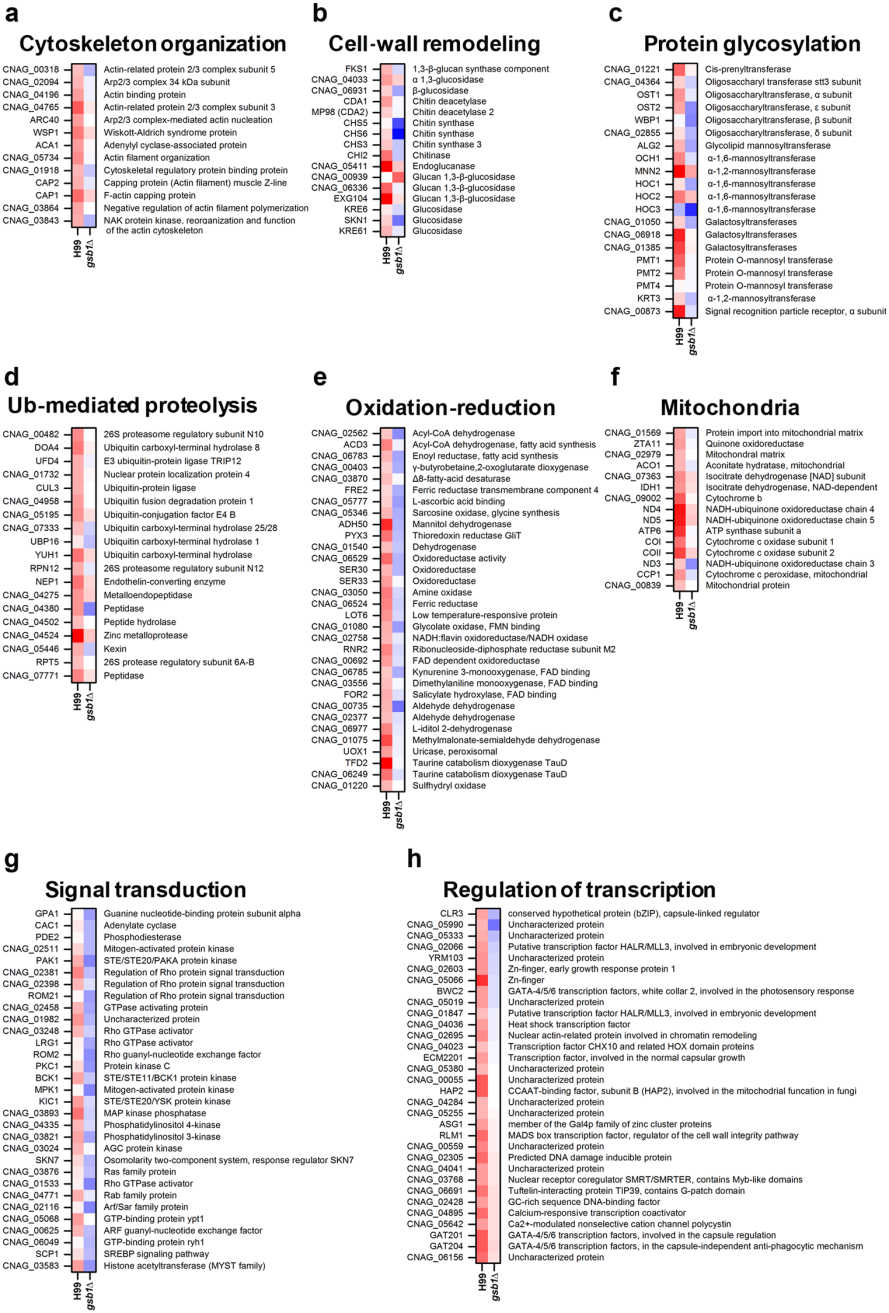
Functional categories of *C. neoformans* genes with differential expression patterns between the WT and *gsb1*Δ strains under oxidative stress. For RNA-Seq analysis under oxidative stress, the WT and *gsb1*Δ strains were inoculated at OD_600_ = 0.25 in YPD containing 1 mM H_2_O_2_. The functional categories of *C. neoformans* genes, which were highly induced in WT but less induced or repressed in *gsb1*Δ, are shown. (**a**) Cytoskeleton organization, (**b**) Cell-wall remodeling, (**c**) Protein glycosylation, (**d**) Ubiquitin (Ub)-mediated proteolysis, (**e**) Oxidation–reduction, (**f**) Mitochondria.

To understand how Gsb1 regulates anti-oxidative-stress responses and adaptation, we carried out additional transcriptome analysis after treatment with 1 mM H_2_O_2_ for 1 h and compared the differentially expressed genes between the WT and *gsb1*Δ mutants. It was previously reported that hydrogen-peroxide-induced oxidative stress causes transient cytoskeletal actin depolarization and causes vacuole fragmentation in *Saccharomyces cerevisiae*, which are mediated by the MAP kinase Slt2^29^. Our RNA-Seq data showed that many genes associated with cellular responses to oxidative stress, such as actin depolymerization, cell-wall remodeling, protein glycosylation, and ubiquitin-dependent proteolysis, were also highly induced in the WT *C. neoformans*, but not in the *gsb1*Δ mutant (Fig. 4a–d). We also observed that several genes involved in oxidation and reduction, mitochondrial function, and removal of mitochondrial ROS, such as cytochrome c peroxidase (*CCP1*)^30^, were not induced in the *gsb1*Δ mutant (Fig. 4e,f). Moreover, a number of genes involved in signal transduction, particularly Ras and cAMP signaling pathways, and some genes encoding for TFs, such as *RLM1*, *HAP2*, *GAT201*, and *GAT204* (Fig. 4g,h) were significantly differentially expressed between the *gsb1*Δ and WT strains. In contrast, genes associated with protein folding and amino acid metabolism were downregulated in the WT strain, but not in the *gsb1*Δ strain under oxidative stress (Supplementary Fig. S5). The transcriptome data strongly indicated that the remarkable hypersensitivity of *gsb1*Δ mutant to oxidative stress results from defective expression of a number of genes involved in antioxidant defense.

### Gsb1 is involved in cell cycle and mating in *C. neoformans*

**Figure 5:**
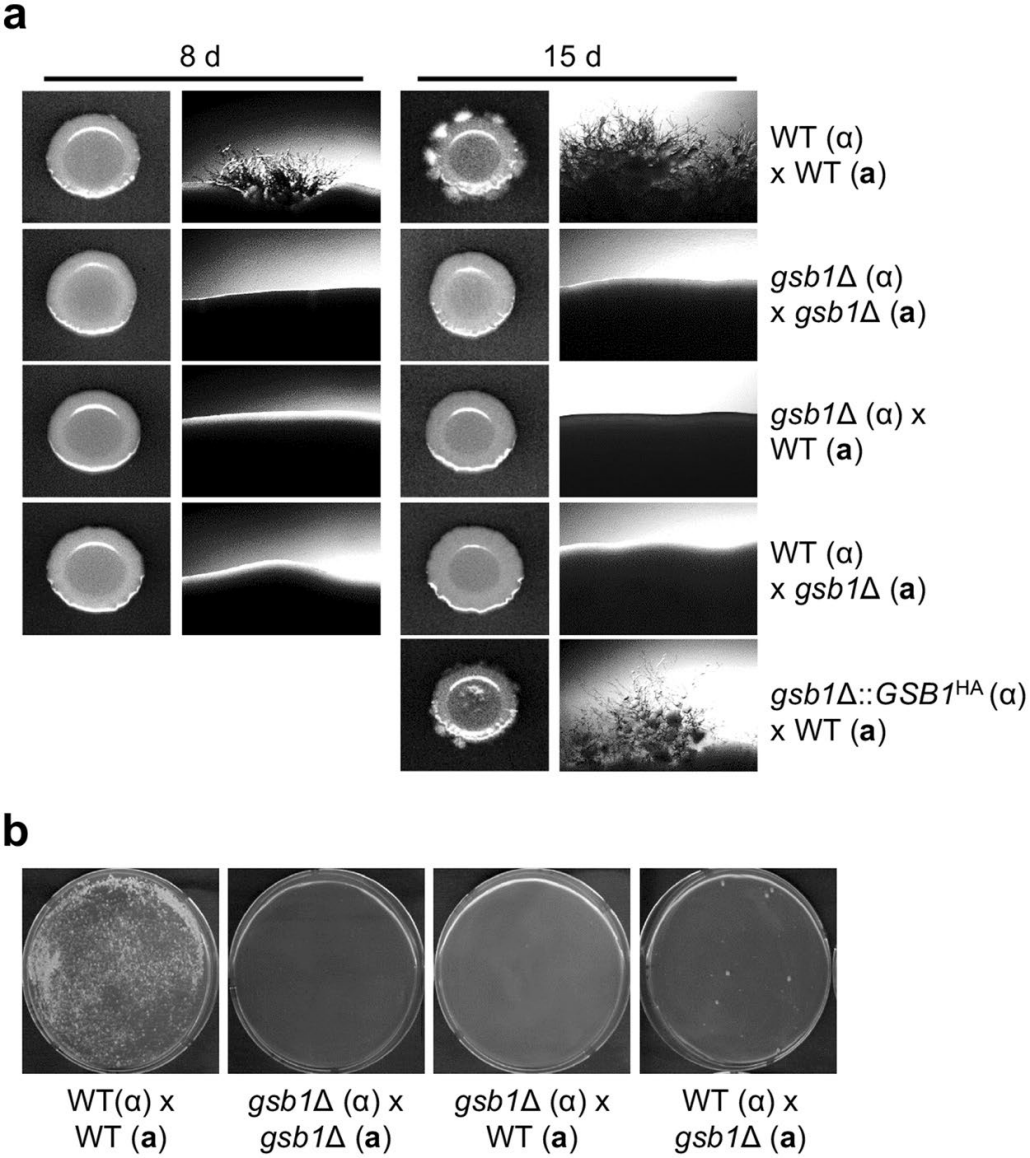
Sexual differentiation analysis of the *gsb1*Δ strains. (**a**) Mating assay. *C. neoformans* cells were initially grown in YPD medium for 16 h at 30 °C. For mating, equal numbers of *MAT*α and *MAT* **a** cells (10^7^ cells/mL) were mixed, spotted on V8 mating medium (pH 5), and incubated in the dark at room temperature. Filamentous growth was photographed using an Axio Scope Z1 microscope equipped with an AxioCam MRm (Carl Zeiss) and scanned. (**b**) Mating cell fusion assay. Cell fusion efficiency was analyzed relative to the control strains [WTα (*NAT*) × WT**a** (*NEO*)] as previously described^32^. The number of cell fusion colonies carrying the selective markers *NAT* and *NEO* was determined after 4 days of incubation at room temperature.

The GO biological process analysis of RNA-Seq data under the normal growth condition indicated that the basal expression of several genes involved in mating-related signaling pathway such as pheromone mitogen-activated protein kinase (MAPK) pathway and cAMP-PKA pathway, including CNAG_00125 (*CRG1*), CNAG_02531 (*CPK1*), CNAG_00179 (*GPA2*), CNAG_00125 (*CRG1*), and CNAG_01855 (*GPR2*)^31^, was significantly decreased in the *gsb1*Δ mutant than in WT. In addition, the basal expression of a TF gene involved in mating (CNAG_03366, *ZNF2*) and a gene coding for a membrane protein (CNAG_05866, *PRM1*) was about two-fold decreased in the *gsb1*Δ mutant than in WT (Supplementary Table S4). The lower expression of these mating-related genes in the *gsb1*Δ strain under normal condition and during mating process was also confirmed by semi-quantitative RT-PCR analysis (Fig. S6). These results led us to examine whether Gsb1 functions are associated with sexual development in *C. neoformans*. First, we constructed an additional *gsb1*Δ mutant on the serotype A *MAT* **a** KN99 genetic background. The *MAT* **a** *gsb1*Δ mutant exhibited a growth rate and stress sensitivity equivalent to those of the *gsb1*Δ mutant on the *MAT*α H99 background (data not shown). The *gsb1*Δ mutants did not form mating hyphae in unilateral [WT (α or **a**) × *gsb1*Δ (**a** or α)] or bilateral [*gsb1*Δ **a** × *gsb1*Δ α] mating processes on V8 medium, and reintroduction of the WT *GSB1* gene completely restored the mating defect in the *gsb1*Δ mutants (Fig. 5a). These results demonstrated that Gsb1 was necessary for mating processes in *C. neoformans*. We also analyzed the cell-fusion efficiency of the Nat^r^ and Neo^r^ control strains (YSB119: *MAT*α, Nat^r^, and YSB121: *MAT* **a**, Neo^r^) and the *gsb1*Δ mutants (*MAT* **a** and *MAT*α) as previously described^32^. No Nat^r^ Neo^r^ dikaryotic cell fusion products were detected from bilateral pairing between the *gsb1*Δ **a** and *gsb1*Δ α mutant strains. Moreover, cell fusion products were hardly detected from unilateral mating, although a few colonies were generated (Fig. 5b). These results indicated that Gsb1 is required for the initial cell-to-cell fusion processes during mating.

**Figure 6:**
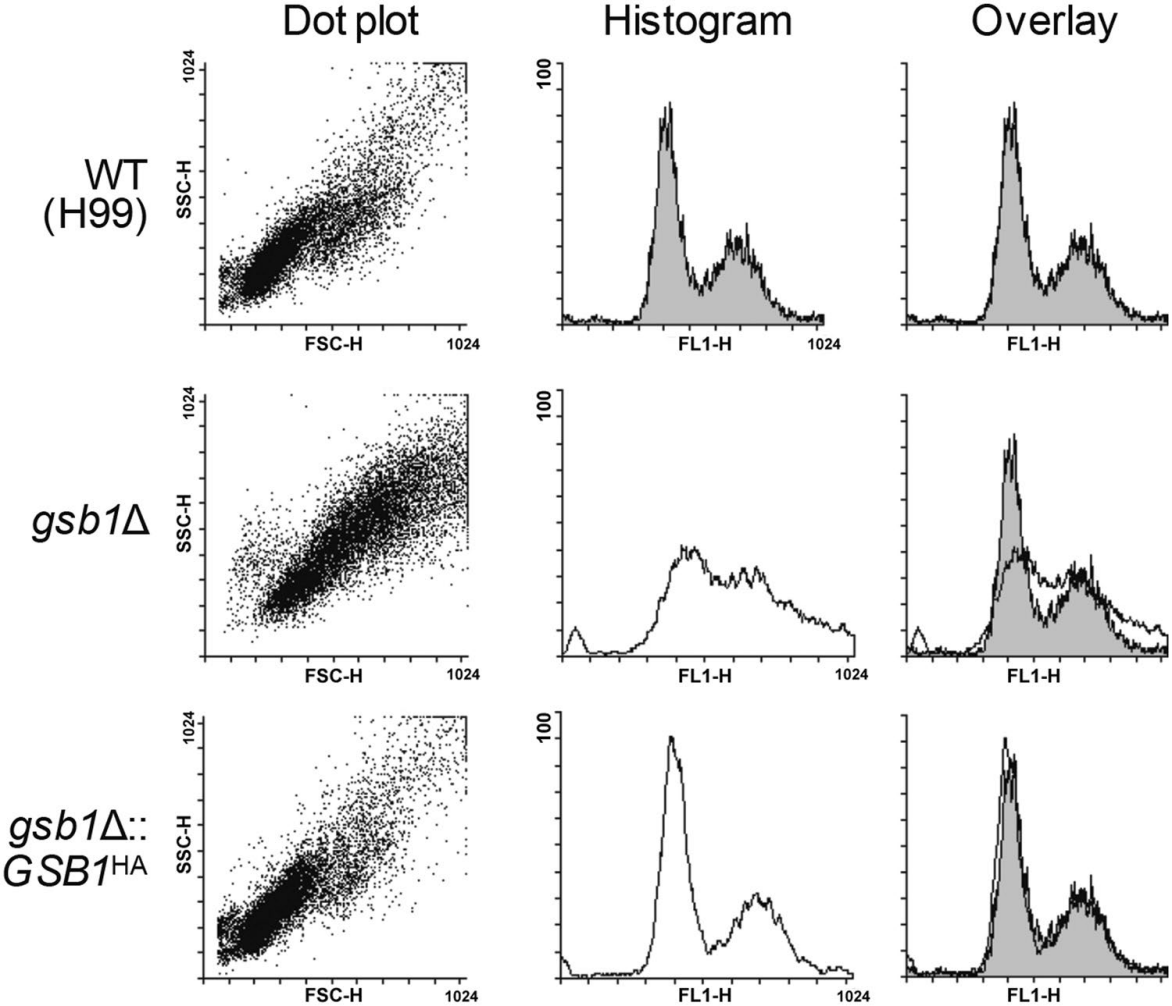
Analysis of cell-cycle progression in *C. neoformans gsb1*Δ. The WT and *gsb1*Δ strains were inoculated at OD_600_ = 0.25 in YPD broth and harvested at OD_600_ = 0.5 (early exponential phase). Flow cytometry was performed using the WT (H99), *gsb1*Δ, or *gsb1*Δ*::GSB1* ^HA^ cells stained with SYTOX. Each dot plot and histogram, and overlay histogram, was plotted representing 10,000 cell events.

Another interesting point revealed from the RNA-Seq data was the increased expression of several genes involved in the cell cycle, such as CNAG_01037 (meiotic recombination), CNAG_07758 (meiotic cell cycle), CNAG_05771 (DNA checkpoint), and CNAG_02658 (regulation of cyclin-dependent serine/threonine kinase) in the *gsb1*Δ mutant under the normal growth conditions (Supplementary Table S3). To monitor the cell cycle in *C. neoformans*, individual cells at the exponential growth phase were stained using the nuclear stain SYTOX Green and analyzed by flow cytometry. Flow cytometry results indicated abnormal cell cycle in the *gsb1*Δ mutant, which exhibited a decreased cell population in G1/G2 phase while an increase in the S phase under normal growth conditions (Fig. 6). These results indicated that the function of Gsb1 protein may affect the G1–S cell cycle in *C. neoformans*.

### Gsb1 is necessary for full virulence of *C. neoformans*

**Figure 7:**
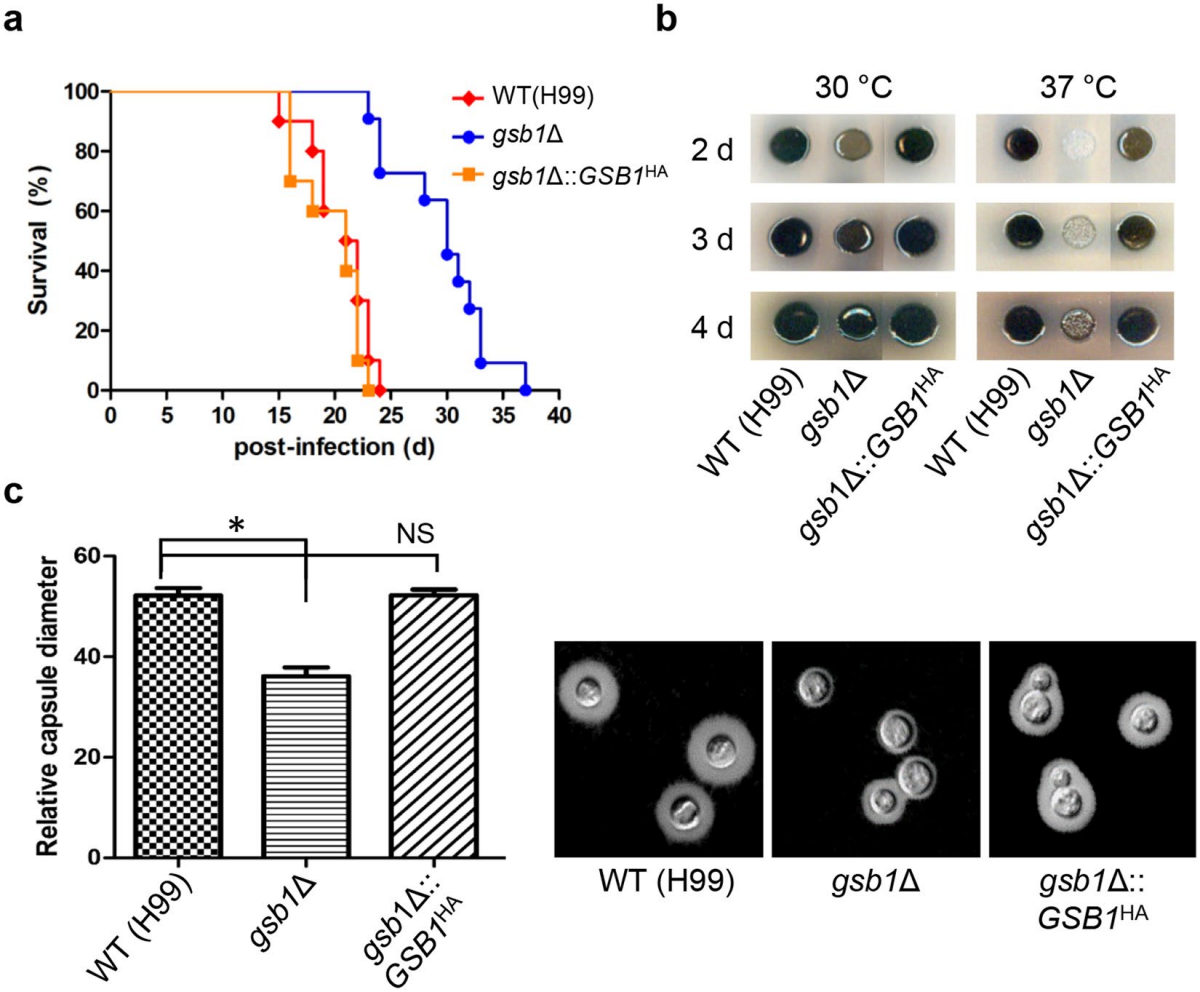
Virulence attributes of *C. neoformans gsb1*Δ mutant strain. (**a**) Virulence analysis of *gsb1*Δ strain. A/JJmsSlc mice were infected with 1 × 10^5^ cells of WT (H99, red diamond), *gsb1*Δ (blue circle), or *gsb1*Δ::*GSB1* ^HA^ complemented strains (orange square) by intranasal instillation. Percent survival (%) was monitored during 6 weeks after infection. P < 0.0001 for WT vs. *gsb1*Δ mutant and P = 0.3406 for WT vs. *gsb1*Δ::*GSB1* ^HA^ complemented strain. (**b**) Melanin analysis. To examine melanin production, cells were spotted on melanin-inducing medium containing l-dihydroxyphenylalanine (100 mg/L) and 0.1% glucose or 1% glucose, and incubated at 30 °C or 37 °C in the dark for 5 days. (**c**) Capsule analysis. Fresh cells on YPD medium were cultured in Sabouraud dextrose medium at 28 °C overnight, washed in 50 mM MOPS buffer (pH 7.3), and inoculated into diluted (1/10) Sabouraud dextrose medium buffered with 50 mM MOPS buffer (pH 7.3) for further incubation at 28 °C or 37 °C for 24 h. Capsule was stained using India Ink (Becton, Dickinson and Company, USA) and visualized using an Axio Scope Z1 microscope equipped with an AxioCam MRm (Carl Zeiss). *P < 0.001 for WT vs. *gsb1*Δ and NS, not significant (P > 0.001) for WT vs. *gsb1*Δ::*GSB1* ^HA^.

The growth defects in the *gsb1*Δ mutant and the overall reduced resistance to diverse environmental stresses, such as heat, and oxidative and nitrosative stresses, prompted us to investigate the *gsb1*Δ virulence in a murine model of systemic cryptococcosis established in A/J mice infected by nasal instillation. We found that the *gsb1*Δ mutant had attenuated virulence compared to the WT strain (H99) (Fig. 7a). Virulence was fully restored in the *gsb1*Δ*::GSB1* ^HA^ complemented strain. This result indicated that Gsb1 is necessary for full virulence in mice. Next, we investigated the roles of Gsb1 in the production of other virulence factors such as the antioxidant melanin and the antiphagocytic capsule in *C. neoformans*. The *gsb1*Δ mutant showed delayed melanin production in L-DOPA medium compared to the WT and *gsb1*Δ*::GSB1* ^HA^ complemented strains, and this defect was more pronounced at 37 °C than at 30 °C (Fig. 7b). Furthermore, two independent *gsb1*Δ mutants were shown to be defective in capsule biosynthesis based on both qualitative and quantitative measurements (Fig. 7c). These results suggest that Gsb1 controls melanin and capsule synthesis in *C. neoformans*.

### Gsb1 is localized throughout the cell under normal conditions but accumulates exclusively in the nucleus upon oxidative stress

**Figure 8:**
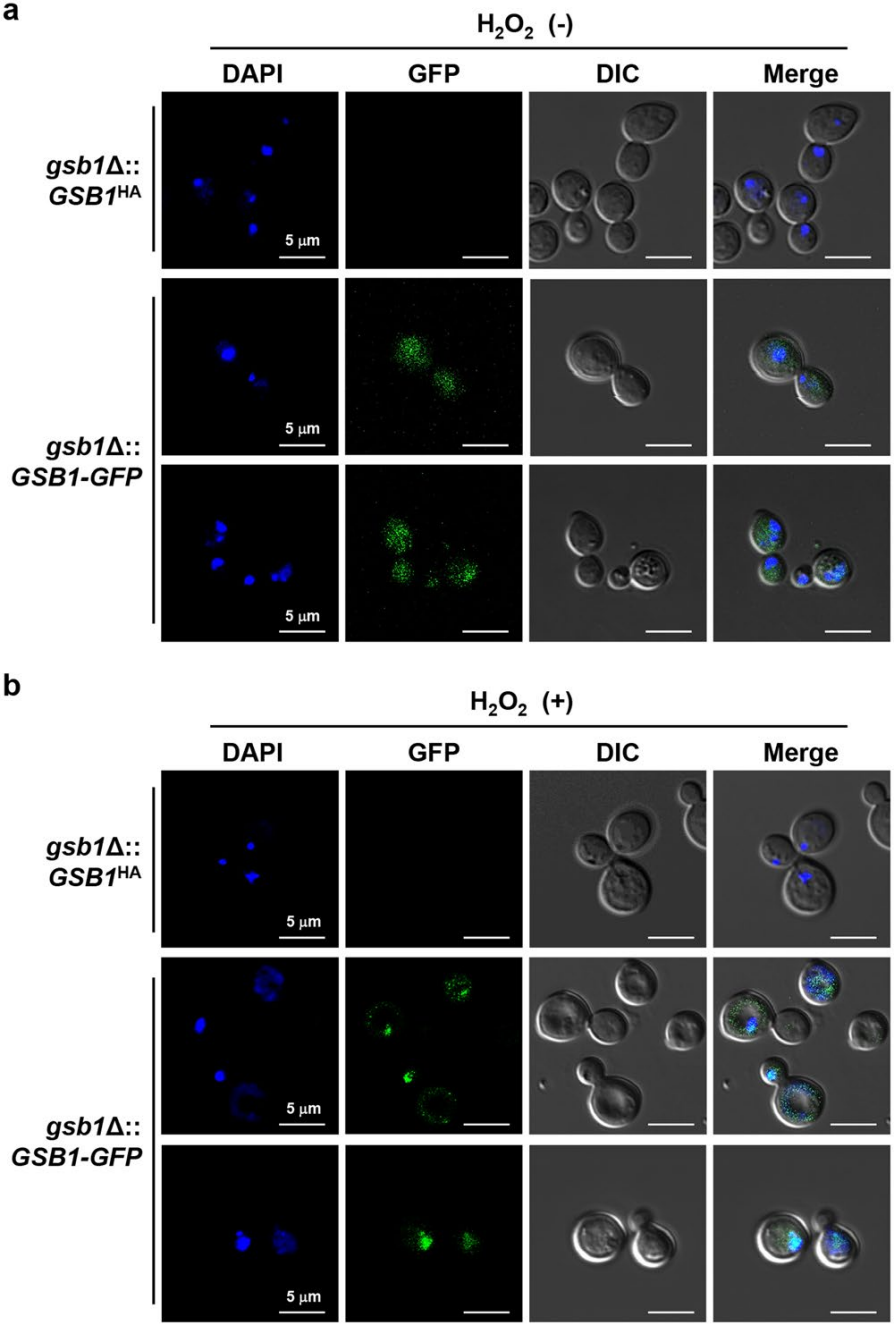
Subcellular localization of Gsb1. (**a**) Under non-stressed condition and (**b**) under oxidative stress condition. The *gsb1*Δ strains containing the Gsb1–GFP fusion vectors, integrated into the terminator region of the *gsb1*Δ deleted allele, were grown to early log phase in YPD or YPD supplemented with 3.5 mM H_2_O_2_ and subjected to confocal microscopy. Nuclei were stained with DAPI and GFP/DAPI merged images were processed by ZEN2011 software (Zeiss).

The transcriptional activity of a bZIP protein can be regulated by multiple mechanisms, including phosphorylation, homo- or hetero-dimerization, or subcellular localization^14, 33^. Gsb1 contains a basic DNA-binding region in the bZIP domain as well as a nuclear export signal (NES) sequence (Supplementary Fig. S3a). For subcellular localization analysis, Gsb1 fused with GFP at its C-terminal (Gsb1-GFP) was expressed under the control of its native promoter and terminator in the background of the *gsb1*Δ mutant strain. The Gsb1–GFP protein could fully restore the growth and stress resistance phenotypes of the *gsb1*Δ mutant (data not shown), implying that the Gsb1–GFP fusion protein was functional *in vivo*. *C. neoformans* cells in the early exponential phase, cultivated under nonstressed conditions or under oxidative stress induced by 3.5 mM H_2_O_2_ treatment for 1 h, were analyzed by a confocal microscope. The *gsb1*Δ strain expressing the Gsb1–GFP fusion construct (*gsb1*Δ::Gsb1–GFP) exhibited green fluorescent signals throughout the cytoplasm and the nucleus (Fig. 8a). We observed that the green fluorescent signals in the *gsb1*Δ::Gsb1–GFP strain became more condensed at the nucleus by H_2_O_2_ treatment (Fig. 8b; Supplementary Fig. S7), indicating that subcellular localization of Gsb1 is modulated by stress signals. We may speculate that despite the presence of NES, some portion of Gsb1 proteins should remain in the nucleus for its basal roles in cell growth under normal conditions, while most portions of Gsb1 proteins should be localized into the nucleus under oxidative conditions to play key roles in the cellular defense responses by *C. neoformans*.

## Discussion

The ability of an organism to survive and proliferate under various environmental conditions requires many defense responses induced mostly at the transcriptional level. Through a variety of stress-activated signaling cascades, activation of different subsets of target genes in response to different environmental stimuli is regulated by specific TFs. The bZIP proteins are widely found as TFs in eukaryotes and display an array of specific functions. The 12 putative bZIP TFs, which have been analyzed for their function by deletion mutation in *C. neoformans*, include Yap1, Yap4, Yap2, Atf1, HapX, Hxl1 (Bzp1), Bzp3, Bzp4, Bzp5 (CNAG_07940), Clr3 (CNAG_00871), Clr4, and Bdr1 (Supplementary Table S6). In this study, we report a novel bZIP protein, Gsb1, as a TF involved in several cellular processes, including stress responses, sexual development, and virulence in *C. neoformans*. The sequence comparison of the bZIP domain of Gsb1 with those of previously identified ATF/CREB proteins from other organisms as well as those in yeast—Gcn4, Yap1, and Cys3—revealed that the bZIP domain of Gsb1 (N-X7-R/K-X6-L-X6-L-X6-L) carries a lysine residue characteristic of ATF1/CREB1 bZIP family (Supplementary Fig. S8a,b). This indicates that Gsb1 might be closely related to the ATF/CREB bZIP family that contains AP-1 like TFs, such as yeast Yap4^34^. However, no known *S. cerevisiae* homologs were retrieved through Blastp analysis using the entire protein sequence or only the bZIP sequence of Gsb1 as a query, except quite a few homologs in some basidiomycetous fungi. Despite the overall low similarity to Gsb1, *C. albicans* Rca1, a TF of the CO_2_-sensing pathway^35^, showed 36% identity only in its bZIP region (Fig. S8c). Thus, we tested whether Gsb1 is a regulator of carbonic anhydrase (CA) genes, such as *CAN1* (a minor CA) or *CAN2* (a major CA), in *C. neoformans* ^5^, but the expression of *CAN2* did not change in the *gsb1*Δ mutant under ambient air condition (data not shown), indicating that Gsb1 is not involved in the CO_2_-sensing mechanisms.

It is notable that the *gsb1*Δ mutant was highly sensitive to both high (39 °C) and low (16 °C) temperatures (Supplementary Fig. S1b). Recent studies have linked the oxidative stress responses in adaptation to low temperature, proposing that a suboptimal growth temperature raises the intracellular ROS levels and induces antioxidant responses^36^. When we deleted *GSB1* in a mutant lacking *ATF1*, encoding an oxidative-stress-responsive TF^16^, the resultant *gsb1*Δ *atf1*Δ double mutant did not show any enhanced sensitivity to oxidative stressors on YPD plate compared to the single *gsb1*Δ mutant strain. Only a subtle cumulative effect of the double mutation on the sensitivity to oxidative stress was observed under a culture condition using a synthetic medium (Supplementary Fig. S9). Such a minor effect of the deletion of *ATF1* strongly suggested that Gsb1 may play more dominant roles in oxidative stress responses and adaptation of *C. neoformans*. This is in good agreement with the attenuated virulence of the *gsb1*Δ mutant strain (Fig. 7), whereas deletion of *ATF1* did not cause a significant difference in the virulence^15^. Loss of another previously characterized oxidative-stress-responsive TF, Yap1, had no significant effect on virulence^15^. Altogether, the results strongly suggest that Gsb1 is a major TF for oxidative stress responses in *C. neoformans*.

**Figure 9:**
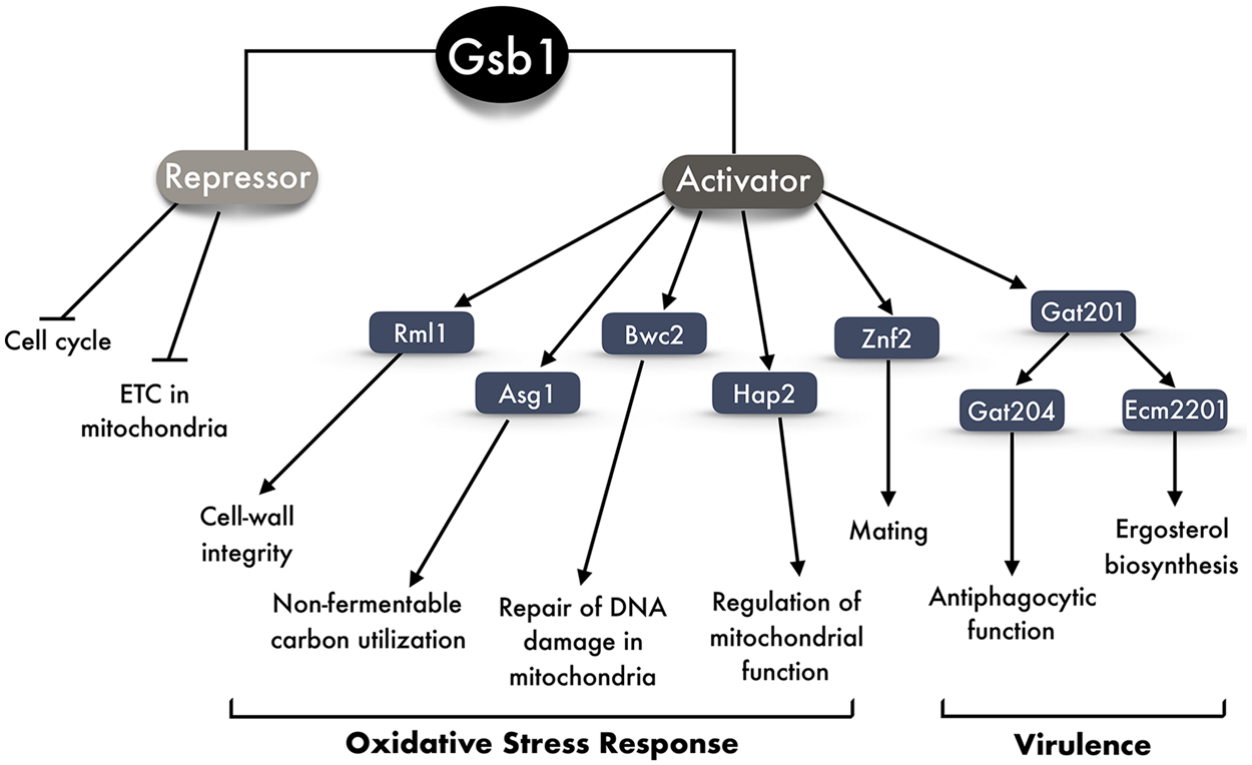
Proposed model of Gsb1’s regulatory roles in *C. neoformans*. Gsb1 is proposed to act as either activator or suppressor of other TFs involved in several cellular processes, such as cell-wall integrity, non-fermentable carbon utilization, mitochondrial homeostasis and function, antiphagocytosis, which are known to be associated with virulence in *C. neoformans*.

Yeast cells respond to ROS by altering the expression of genes encoding antioxidant defense mechanisms and genes encoding enzymes, which detoxify ROS and repair the resultant cellular damage. Previous studies in *S. cerevisiae* reported that MAPK and other signaling pathways (PKC1-MAPK, TOR, Ras/PKA-cAMP) are involved in the oxidative stress responses to maintain cellular redox homeostasis. The stress-activated signaling cascades are shown to involve the activation of a number of TFs, such as Yap1, Skn7, Rlm1, Msn2/Msn4, and Sfp1, which are required for the expression of a subset of genes counteracting the oxidative stresses^37^. In addition, the cellular responses to oxidative stress involve MAPK-dependent feedback regulation of the actin cytoskeleton, a known target for oxidation^29^. Our RNA-Seq-based transcriptome profiling of the *gsb1*Δ mutant compared with the WT strain in *C. neoformans* revealed that diverse groups of genes appeared to be regulated by Gsb1 under oxidative stress conditions, but a considerable number of genes were also subject to Gsb1 regulation under nonstressed conditions (Figs 3 and 4). Notably, we propose that Gsb1 acts as either activator or suppressor of other TFs (Fig. 9): Rlm1 for cell-wall integrity, Asg1 for non-fermentable carbon utilization, Bwc2 for light sensing, Hap2 for mitochondrial homeostasis and function, Gat201 and Gat204 for antiphagocytic functions, and Ecm2201 for ergosterol biosynthesis. Most of these Gsb1-regulated TFs were known to be associated with virulence in a previous study on model-driven mapping of *C. neoformans* transcriptional network^38^. In contrast, Gsb1 may act as activator of the mating process but also as a repressor of cell cycle and mitochondrial ETC to maintain cellular homeostasis under normal cell growth. TFs containing the bZIP domain typically bind DNA as dimers, and the formation of bZIP homo- and heterodimers provides huge combinatorial flexibility during the regulation of transcription^14^. Considering that Gsb1 is involved in inducing or repressing the expression of such diverse sets of genes, it can be speculated that Gsb1 might interact with other bZIP factors to form heterodimers with diverse DNA-binding specificities.

Several bZIP TFs were reported as nucleocytoplasmic shuttling proteins, whose subcellular localizations were shown to respond to oxidative stress. The oxidative-stress-specific TFs, such as Yap1 in *S. cerevisiae* and Pap1, a Yap1 ortholog in *Schizosaccharomyces pombe*, were found primarily in the cytoplasm under normal conditions but present in the nucleus only in the presence of oxidative stress^39, 40^. Our data on the subcellular localization of Gsb1 supported the role of Gsb1 as a TF whose function is required not only for inducing the oxidative stress responses but also for maintaining the normal growth of *C. neoformans* under nonstressed conditions. Gsb1 was observed to localize throughout the cell under normal conditions, even though it was exclusively accumulated in the nucleus upon oxidative stress (Fig. 8, Supplementary Fig. S7). The activity of TFs is controlled by several post-translational mechanisms, including ubiquitination, proteolytic processing, and proteasomal degradation as well as by its phosphorylation. Previous studies on bZIP TFs, including Pap1 in *S. pombe* ^40^ and Met4 in *S. cerevisiae* ^41^ indicate that ubiquitin-mediated proteolysis or inactivation is a critical regulator to downregulate uncontrolled function of the bZIP TFs for maintaining cellular homeostasis. The possibility that Gsb1 might be also subjected to post-translational mechanisms, such as ubiquitination and phosphorylation, was indicated by the western blot data of Gsb1^HA^ protein, which showed the presence of smeared protein bands with higher molecular weights under oxidative condition but not under the normal growth condition (Supplementary Fig. S3). Several interesting issues remain to be addressed by further studies, including (i) identification of direct target genes whose promoters interact with Gsb1, (ii) investigation of the presence of interacting partners to form heterodimers with Gsb1, (iii) elucidation of the regulatory mechanisms of differential inactivation of the NES sequence of Gsb1 for nuclear localization.

Human fungal pathogens that infect a host from the natural environment, including *C. neoformans*, should have the capacity to cope with oxidative stress conferred by the host innate immune cells. The oxidative and nitrosative stress regulatory mechanisms in *C. neoformans* appear to be complex and may involve multiple TFs. Our results presented in this study highlight a key role for a novel bZIP protein, Gsb1, in coordinating the expression of various sets of genes involved not only in oxidative stress but also in several other cellular processes, such as mating and cell division. Thereby, modulating the activity of Gsb1 to fine-tune regulation of the oxidative stress responses and cellular homeostasis is an attractive approach to developing new antifungal drug candidates.

## Materials and Methods

### Strains, media, plasmids, and primers

The *C. neoformans* strains used in this study are listed in Supplementary Table S1. Yeast cells were maintained and cultured in YPD medium (1% yeast extract, 2% peptone, 2% glucose). Niger seed medium for melanin production, agar-based Dulbecco’s modified Eagle’s medium for capsule production, and V8 medium for mating were prepared as described previously^32^. The plasmids and primers used in this study are listed in the Supplementary Table S2. Information on genomic DNA sequences for each gene was obtained from the *C. neoformans* serotype A genome database (Duke university/Broad Institute of Harvard).

### Disruption of *C. neoformans* genes encoding the putative bZIP proteins

CNAG_00871 (*CRL3*), CNAG_03976, CNAG_07560 (*GSB1*), CNAG_07940 (*BZP5*) on the *C. neoformans* serotype A H99 (*MAT*α) background were disrupted by using double-joint PCR (DJ-PCR) combined with biolistic transformation as described previously^42^. The first 5′-flankng or 3′-flanking region of each ORF obtained by using the H99 genomic DNA was amplified by PCR using the primer sets detailed in the Supplementary Table S2 (C790/C791 and C815/C793 for CNAG_00871, C820/C821 and C822/C823 for CNAG_03976, C824/C825 and C826/C827 for CNAG_07560, C828/C829 and C830/C831 for CNAG_07940). The dominant selectable marker *NAT* (nourseothricin acetyltransferase) was amplified using pNATSTM#225 and the primer sets M13Fe/B1455 and B1454/C814. The 2.03 kb fusion PCR product of 5′-flanking region of *GSB1* (0.72 kb) and a part of *NAT* marker (1.31 kb), and the 1.42 kb fusion PCR product of 3′-flanking region of *GSB1* (0.76 kb) and a part of *NAT* marker (0.66 kb) were generated by overlap PCR using the primer sets C824/B1455 and B1454/C827, respectively, and the combined templates of the first PCR products. Other DJ-PCR disruption cassettes were obtained by the same procedure. The 2.092-kb fusion PCR product of the 5′-flanking region of CNAG_00871 and the 1.31-kb *NAT* marker, and 1.452-kb fusion PCR product of the 3′-flanking region of CNAG_00871 and the 0.66-kb *NAT* marker were generated by overlap PCR using the primer sets C790/B1455 and B1454/C793, respectively. The 2.233-kb product of the 5′-flanking region of CNAG_03976 and the 1.31-kb *NAT* marker, and 1.412-kb product of 3′-flanking region of CNAG_03976 and the 0.66-kb *NAT* marker were amplified using the primer sets C820/B1455 and B1454/C823, respectively. The 2.105-kb product of the 5′-flanking region of CNAG_07940 and the 1.31-kb *NAT* marker, and 1.367-kb product of 3′-flaking region of CNAG_07940 and 0.66-kb *NAT* marker, were amplified using the primer sets C828/B1455 and B1454/C823, respectively. The *NAT* disruption cassettes were biolistically transformed into the H99 strain. Stable transformants were selected on the YPD medium containing nourseothricin (100 μg/mL), initially screened by diagnostic PCR, and confirmed by Southern blotting (Supplementary Fig. S2).

For the *GSB1* disruption on the background of KN99 (*MAT*a) strain, the 0.47-kb of the *GSB1* promoter was cleaved using NheI/KpnI from pJAFS1–GSB1PT vector, which contains its promoter and terminator, and inserted between the SpeI and KpnI sites of pJAF1 vector, generating pJAF1–gsb1DP1. The pJAF1–gsb11DP2 vector harboring the *gsb1*Δ::*NEO* ^R^ disruption cassette was constructed by insertion of the 0.9-kb fragment containing the 3′ part of the ORF with its 3′-UTR region, amplified using primers C991/992, between the EcoRV and XbaI sites of pJAF1–gsb1DP1. The *gsb1*Δ::*NEO* ^R^ disruption cassette (3.3 kb), amplified by PCR using primers C904/C827 from pJAF1–gsb1DP2, was transformed biolistically into KN99**a** (*MAT* **a**) strain. Stable transformants were selected on the YPD medium containing G418 (200 μg/mL) and confirmed by diagnostic PCR and Southern blotting (Data not shown).

### Construction of *GSB1* complementation and Gsb1-GFP fusion vectors

To generate a *GSB1* complementation vector, the 0.49-kb promoter and 0.98-kb terminator fragments of *GSB1* were amplified by PCR using the primer sets C904/C905 and C906/C907, respectively, using the genomic DNA of strain H99; the products were fused by a second PCR reaction using primers C904/C907. The 1.45-kb fusion PCR product was subcloned into pJAFS1 containing the *NEO* selection marker, generating the plasmid pJAFS1–GSB1PT. The untagged *GSB1* ORF amplified using C908/C909 and the C-terminal HA-tagged *GSB1* ORF amplified by two PCR reactions using C908/911 and C908/C912 were subcloned between NheI and NotI sites of pJAFS1–GSB1PT, resulting in pJAFS1–GSB1R1 and pJAFS1–GSB1R3CH, respectively. The vectors were excised at the single MluI site of the *GSB1* terminator and reintegrated into the native *GSB1* locus of the *gsb1*Δ strain by biolistic transformation. To construct a C-terminal Gsb1–GFP fusion vector, the HindIII/BamHI-digested *GSB1* fragment, amplified using primers C1069/C1070, and the BamHI/NotI*-*digested *GFP* fragment, amplified using primers C11071/C1072, were subcloned into the HindIII/NotI sites of pJAFS1–GSB1R3CH, resulting in pJAFS1–GSB1R6CF. The GFP vectors were digested at the MluI site at the *GSB1* terminator and reintegrated into the native *GSB1* locus of the *gsb1*Δ strain.

### Mitochondrial staining

Mitochondria were stained as described previously with slight modifications^24^. Strains grown in YPD medium at initial OD_600_ = 0.3 for 3 h were incubated for 30 min after addition of MitoTracker Red CMXRos (20 nM final concentration, Invitrogen). Cells were washed twice in 1 × phosphate-buffered saline (PBS) buffer and collected by centrifugation at 300 × *g* for 3 min. Then cells were fixed in 3.7% formaldehyde in a rotator at 25 rpm for 10 min and washed three times in 1 × PBS buffer three times. For 4′,6-diamidino-2-phenylindole (DAPI) staining, an equal volume of cell suspension and PBS buffer containing DAPI (5 μg/mL, Invitrogen) were mixed. Sections were mounted on slides in an antifade medium (Invitrogen) and examined using an inverted confocal microscope (Zeiss LSM700/Axio Scope Z1) equipped with a transmitted light detector T-PMT (Carl Zeiss).

### RNA-Seq analyses

Total RNA was extracted as previously described^13^. Single-stranded cDNA was synthesized using an RnaUsScript reverse transcriptase kit (LeGene Biosciences). RNA sequencing was performed as previously reported using Illumina HiSeq2500^43^. Quality-filtered sequence reads were mapped to the reference genome sequence of *C. neoformans* var. *grubii* H99 (RefSeq assembly accession number GCF_000149245.1) using the CLC Genomics Workbench 5.5 (CLC bio). The relative transcript abundance was calculated by counting the Reads Per Kilobase of exon model per Million mapped reads, RPKM^44^. The processed RNA-seq ORFs were matched with 4,296 annotated gene products from GOProfiler (https://omictools.com/goprofiler-tool) for taxonomy 235443 *C. neoformans* var. *grubii* H99 and analyzed by GOSlimViewer using the Generic GOSlim Set developed by the GO Consortium. Gene ontology (GO) analysis was performed using tools by AgBase^45^. Processed data were deposited in the Gene Expression Omnibus (GEO) database with accession number GSE96543.

### Flow cytometry

For flow cytometry, *C. neoformans* cells were stained using the SYTOX Green as described previously^46^. Yeast cells were fixed in 70% ethanol with slow mixing for 1 h at RT, washed in 1 mL distilled water, and incubated in 0.5 mL RNase solution (2 mg/mL in 50 mM Tris-HCl, pH 8.0, 15 mM NaCl, used after boiling for 15 min) for 3 h at 37 °C. Cells were then collected, incubated in 0.2 mL protease solution (5 mg/mL pepsin, 4.5 μL/mL HCl) for 30 min at 37 °C. After centrifugation, yeast cells (5 × 10^7^ cells/mL) were resuspended in 50 mM Tris-HCl, pH 7.5. The cell suspension (25 μL) was mixed with 1 mL SYTOX Green solution (1 μM SYTOX Green in 50 mM Tris-HCl, pH 7.5; Molecular Probes), sonicated briefly, analyzed on a FACSCalibur System (BD Biosciences) at 488-nm excitation. The SYTOX fluorescence data were analyzed using Flowing software 2.5.1 (Cell Imaging Core, Turku Centre for Biotechnology, Finland).

### Virulence analysis

Capsule and melanin production levels were measured as described previously^47, 48^. Animal studies were conducted in Chung-Ang University Animal Experiment Center III facility, and were approved by the Ministry of Food and Drug Safety (MFDS, Korea). All experiments were performed in accordance with relevant guidelines and regulations. Six-week-old female A/JJmsSlc mice (Japan SLC, Inc., 18–22 g) were used in this study. For infection, strains were cultured in YPD medium overnight at 24 °C, washed twice in sterile PBS, and resuspended in sterile PBS at 2 × 10^6^ cells per mL. Dilutions of the cells were plated onto YPD medium and incubated at 24 °C for 72 h to determine viability. Ten mice per strain were anesthetized using Zoletil (Virbac Pty. Ltd) and infected via intranasal instillation with 10^5^ cells (in 50 µL). Survival was monitored twice daily, and moribund mice were euthanized using CO_2_. The Kaplan–Meier survival curves were generated using Prism 5.02 (GraphPad Software), and *P* values were calculated using the Mantel–Cox Log-rank test.

## Electronic supplementary material

Supplementary Information
