# Supplementary material for: A novel bZIP protein, Gsb1, is required for oxidative stress response, mating, and virulence in the human pathogen Cryptococcus neoformans
Source: Sci Rep. 2017 Jun 22;7:4044. doi: 10.1038/s41598-017-04290-8 (PMC5481450; doi:10.1038/s41598-017-04290-8)
Supplement: Supplementary file 1 — Supplementary Information [file 41598_2017_4290_MOESM1_ESM.pdf]

Supplementary Information

A novel bZIP protein, Gsb1, is required for oxidative stress response, mating, and virulence in the human pathogen *Cryptococcus neoformans*

Seon Ah Cheon[1,2], Eun Jung Thak[1], Yong-Sun Bahn[3], and Hyun Ah Kang[1*]

[1]Department of Life Science, Chung-Ang University, Seoul 06974, Korea; [2]Center for Fungal Pathogenesis, Seoul National University, Seoul 08826, Korea; [3]Department of Biotechnology, Center for Fungal Pathogenesis, Yonsei University, Seoul 03722, Korea

[*]Correspondence e-mail: hyunkang@cau.ac.kr

Contents

Supplementary Figures S1-S9

Supplementary Tables S1-S6

Supplementary Figure 1

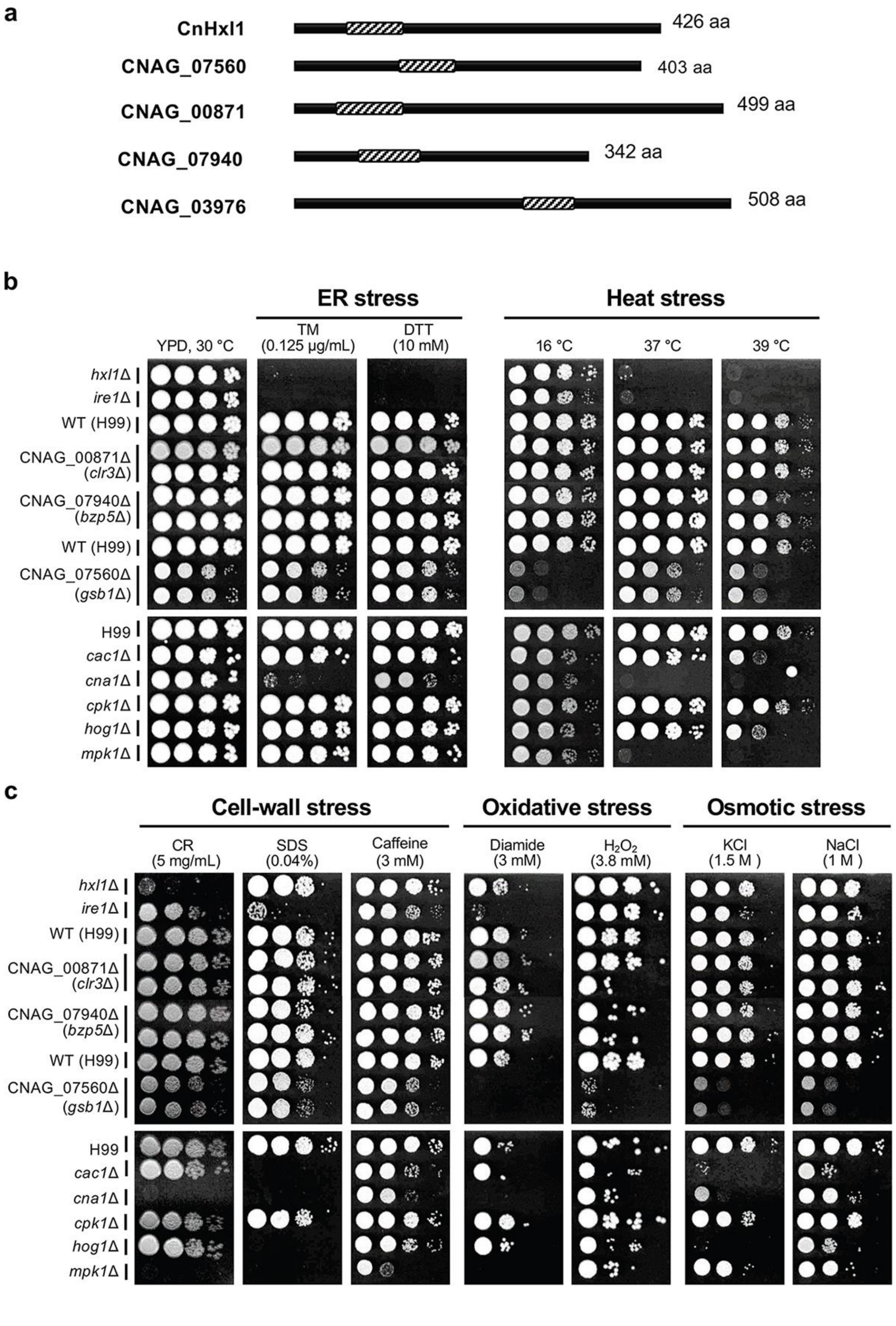

Fig. S1. Identification and comparative analysis of *C. neoformans* putative bZIP proteins. (a) Identification of the *C. neoformans* putative bZIP proteins carrying the bZIP domain with similarity to that of Hac1/Xbp1. The bZIP domain was analyzed using the SMART tool[1]. Amino acid (aa); bZIP domain (hatched box). (b) Growth analysis of the deletion mutants of putative bZIP under various stress conditions. For comparative analysis, the new bZIP deletion mutants constructed in this study (CNAG_00871Δ, CNAG_07940Δ, and CNAG_07560Δ) were spotted with the UPR mutants (*hxl1*Δ and *ire1*Δ) and the oxidative-stress-sensitive mutants (*cac1*Δ, *cna1*Δ, *cpk1*Δ, *hog1*Δ, and *mpk1*Δ), which were previously characterized (Supplementary Table S1). Yeast cells, grown in YPD overnight, were washed in distilled water twice, diluted serially, and spotted on YPD media containing various stressors at the indicated concentrations, and then incubated for 4 days at 30 ℃. For heat stress, *C. neoformans* cells were cultivated at 16 ℃, 30 ℃, or 37 ℃, respectively.

Supplementary Figure 2

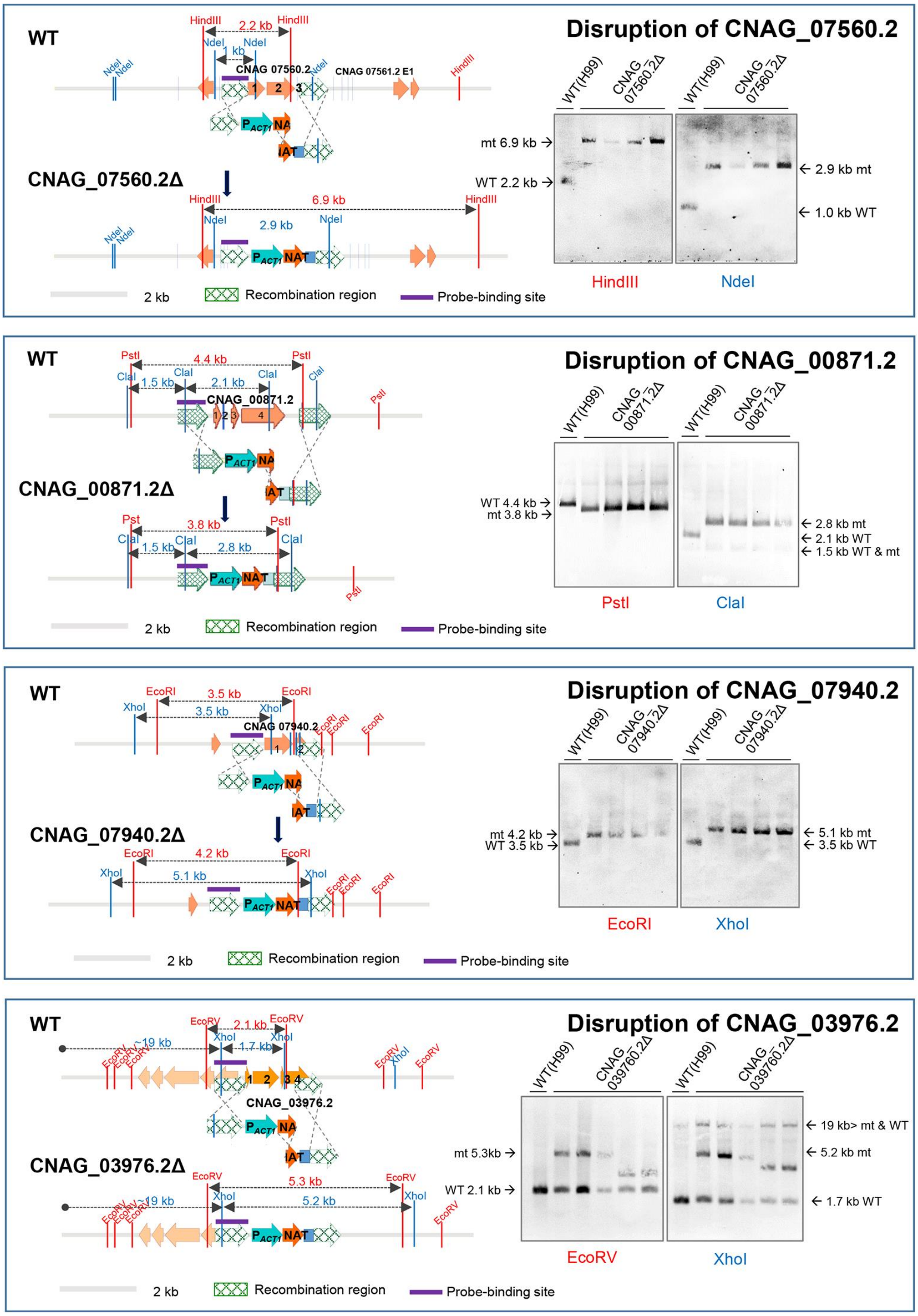

Fig. S2. Disruption of the putative *C. neoformans* bZIP genes and validation by Southern blotting.

Supplementary Figure 3

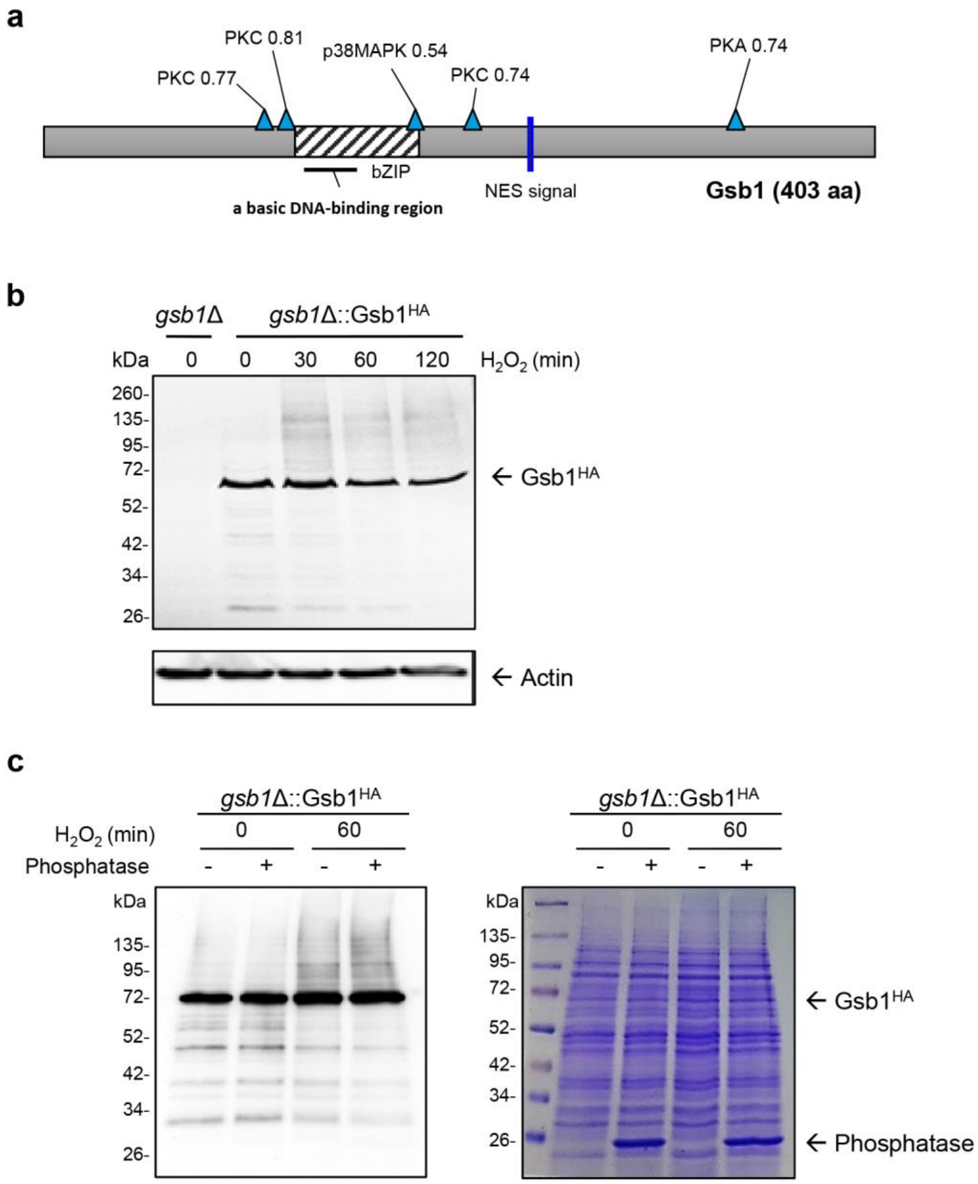

Fig. S3. Structural and expression analyses of *C. neoformans* Gsb1 protein. (a) Domain analysis of HA-tagged Gsb1 protein. The 21 putative phosphorylation sites (18 serine residues, 3 threonine residues) were predicted by using the NetPhosYeast 1.0. The putative kinase phosphorylation sites involved in cell-wall integrity pathway, such as PKC, PKA, and p38MAPK (Hog1), are indicated by arrowheads and a correlation coefficient score. (b) Expression analysis of Gsb1^{HA} protein. *C. neoformans* cells were grown in YPD medium to

early exponential phase, exposed to 3.5 mM H_2O_2, and collected in time-course (0, 30, 60, 120 min). Total proteins were prepared with phospho-protein extraction buffer (50 mM Tris-HCl [pH 7.5], 1% [wt/vol] sodium deoxycholate, 5 mM sodium pyrophosphate, 10 nM sodium orthovanadate, 50 mM NaF, 0.1% [wt/vol] SDS, and 1% [vol/vol] Triton X-100) and analyzed by western blotting with monoclonal rat anti-HA antibody (Roche) and anti-beta actin antibody (ab8224, Abcam) as a loading control. (c) Phosphatase treatment analysis of Gsb1^{HA} protein. Total proteins were extracted, treated with or without protein phosphatase (24 unit/μg of total proteins, New England BioLabs) in the reaction buffer for 1 hr at 30 ℃ as described previously[2], and subjected to western blot analysis. Left panel (western blot), right panel (Coomassie blue staining).

Supplementary Figure S4

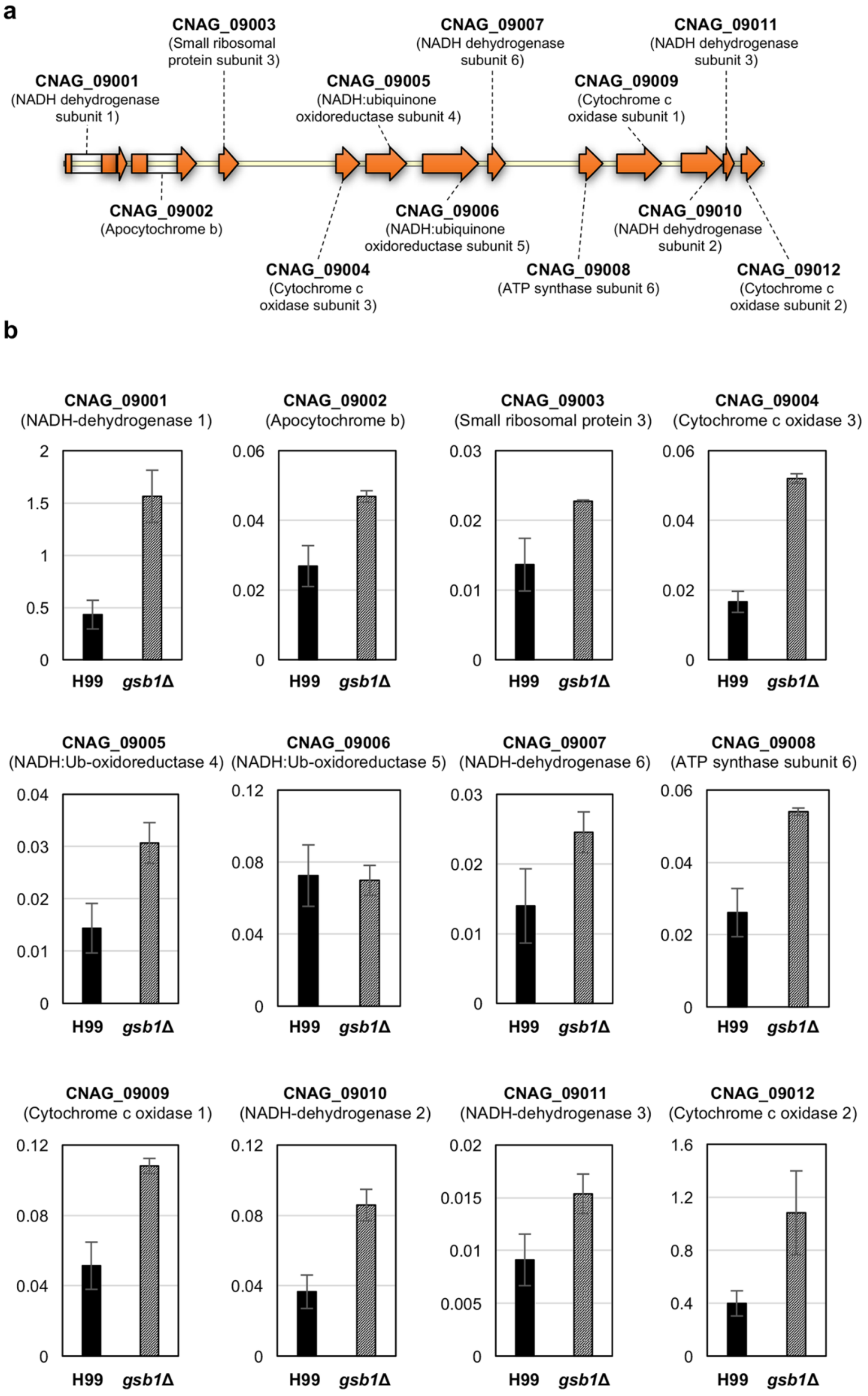

Fig. S4. Validation of up-regulated expression of mitochondrial genes in the *C. neoformans gsb1*Δ strain by quantitative RT-PCR (qRT-PCR) analysis. (a) Structural organization of mitochondrial genome of *C. neoformans* var. *grubii* H99 (Accession number CP003834). (b) qRT-PCR analysis of *C. neoformans* mitochondrial gene expression. Total RNAs were extracted as previous described[3] from the H99 and *gsb1*Δ strains, grown in YPD to early exponential phase. cDNAs were prepared from total RNAs using a RnaUsScript reverse transcriptase (LeGene Biosciences) and subjected to qRT-PCR with the primers for twelve mitochondrial genes listed in Supplementary Table S2. qRT-PCR was performed in duplicate for the mixture of two independent cDNA samples using SYBR Premix Ex Taq (Tli RNaseH Plus) (Takara) and CFX96™ Real-Time PCR detection system (Bio-Rad). Normalized fold expression (ΔΔC(q)) was calculated with the CFX manager software using *GAPDH* as a reference gene.

Supplementary Figure 5

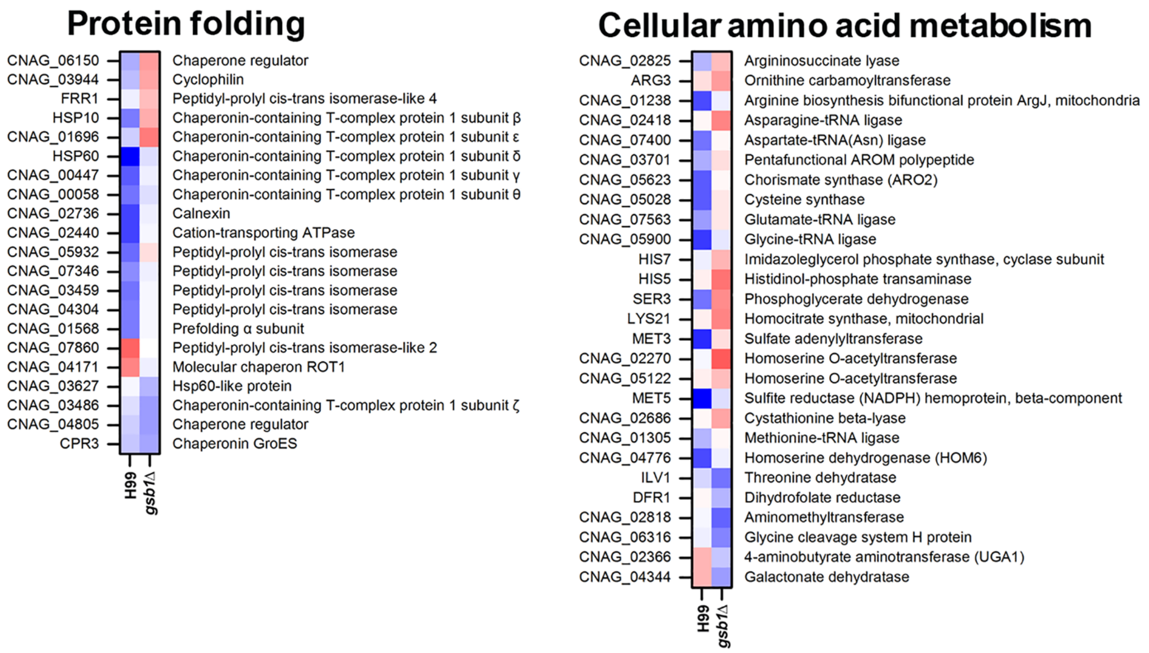

Fig. S5. Representative functional categories of *C. neoformans* genes with more repressed expression in the *gsb1*Δ than in WT under H_2O_2-induced oxidative stress condition. The differential gene expression was analyzed based on RNA-Seq data.

Supplementary Figure 6

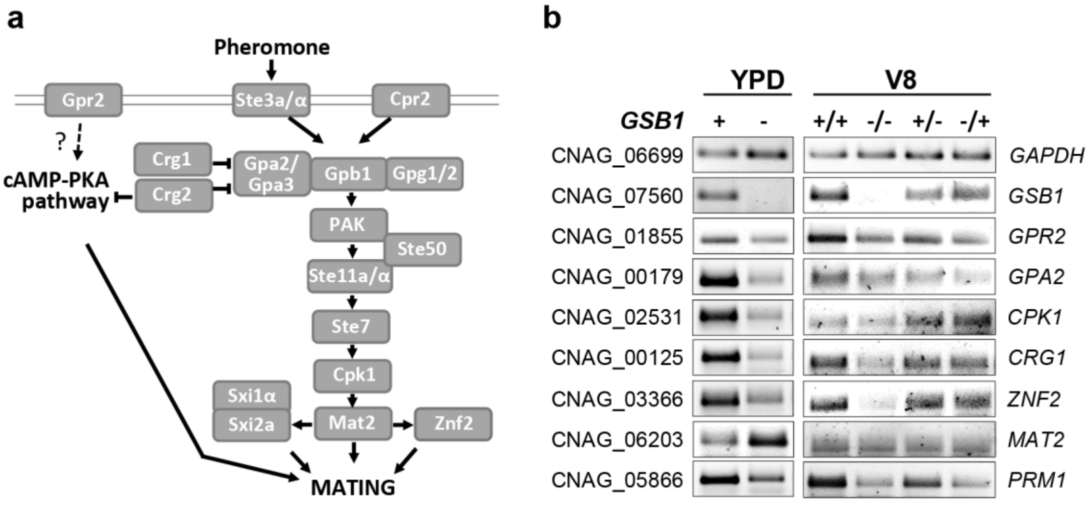

Fig. S6. Expression analysis of genes involved in the mating-related signaling pathway of *C. neoformans*. (a) Proposed pheromone response pathway in *C. neoformans*, which is modified from Kozubowski and Heitman[4]. (b) Semi-quantitative reverse transcriptase-polymerase chain reaction (RT-PCR) analysis of genes associated with the mating-related signaling pathway under normal cultivation and mating conditions. For normal cultivation condition, cDNA was prepared from MATα WT (H99) and MATα *gsb1*Δ strains cultivated upto early exponential phase in YPD. For mating condition, cDNA was prepared from cells cultivated in V8 medium. In detail, the MATα WT (H99), MAT**a** WT (KN99), MATα *gsb1*Δ and MAT**a** *gsb1*Δ strains were grown in YPD medium for 16 h at 30 °C and then washed with distilled water three times. Equal numbers of MATα and MAT**a** cells (5x10^7 cells) were mixed, spreaded on V8 mating medium (pH 5), and harvested after incubation in the dark at room temperature for 20 h. Semi-quantitative RT-PCR was conducted out using serially diluted cDNA with Maxime™ PCR PreMix (i-Taq) (iNtRON Biotechnology) with the primers listed in Supplementary Table S2.

Supplementary Figure S7

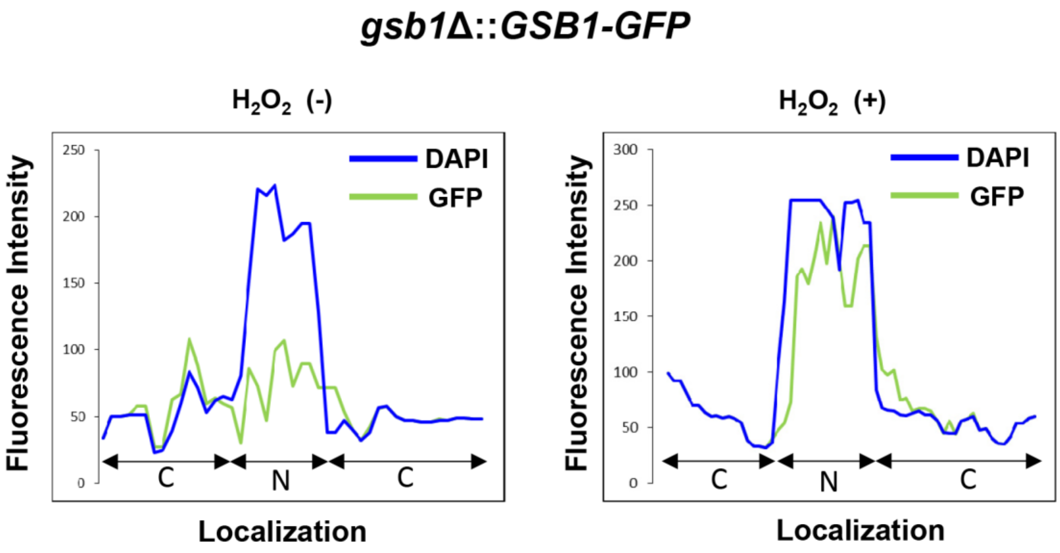

Fig. S7. Representative localization image of Gsb1-GFP protein in *C. neoformans* cultivated without and with H_2O_2. Images of Gsb1-GFP protein expressed in *C. neoformans* cells (Fig. 8) were analyzed using NIS-Elements image analysis software (Nikon), and one of representative images was shown (C: cytoplasm, N: Nucleus).

Supplementary Figure 8

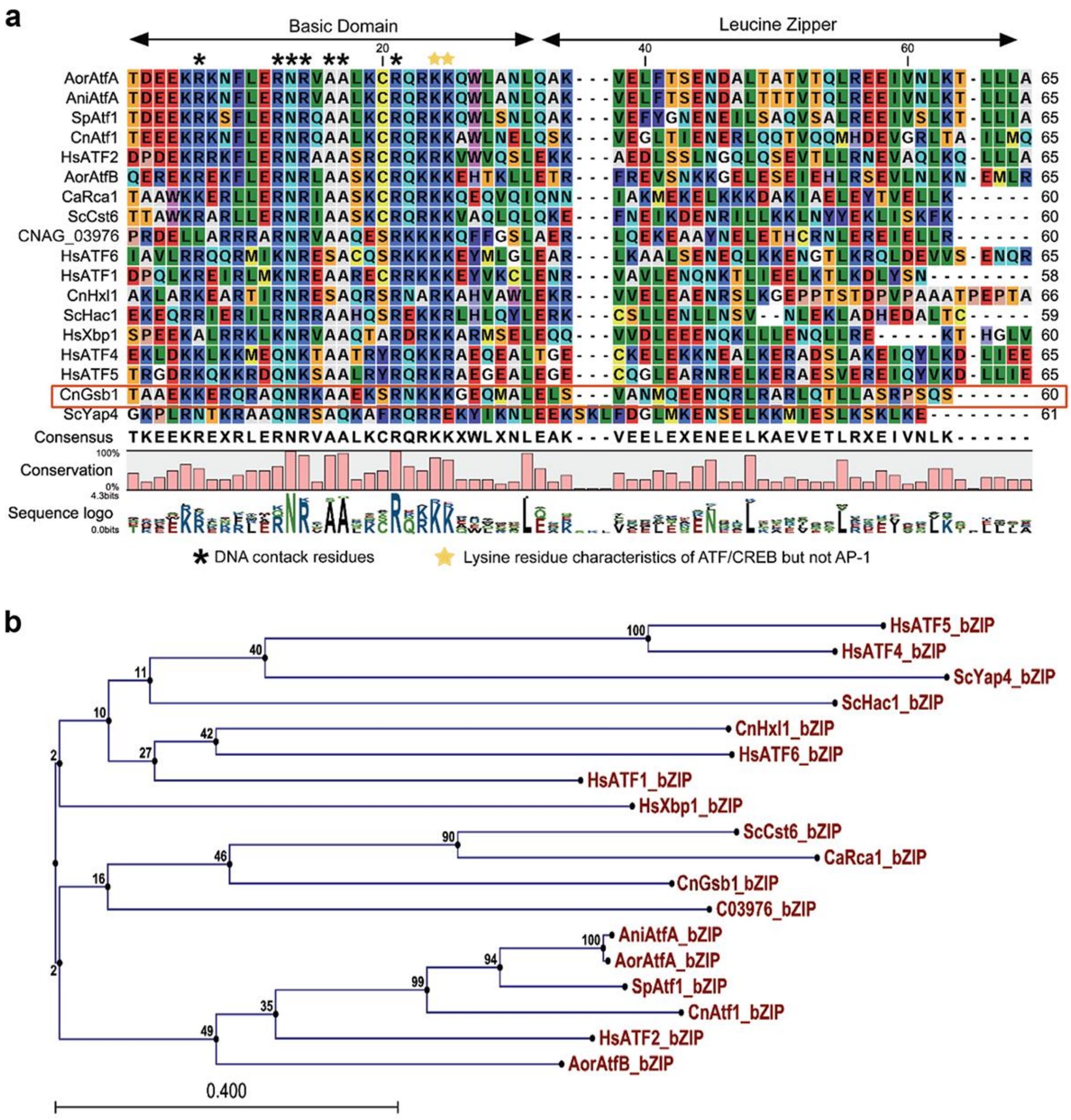

c

	ScCst6_bZIP	CNAG_03976_bZIP	CnGsb1_bZIP	CaRca1_bZIP	CnHxl1_bZIP	CnAtf1_bZIP	
ScCst6_bZIP		25	30	48.3	21.7	36.7	ScCst6_bZIP
CNAG_03976_bZIP	195		26.7	26.7	25	21.7	CNAG_03976_bZIP
CnGsb1_bZIP	159.9	183.8		36.7	28.3	30	CnGsb1_bZIP
CaRca1_bZIP	84.4	183.8	125		18.3	33.3	CaRca1_bZIP
CnHxl1_bZIP	227	195	171.1	268		23.1	CnHxl1_bZIP
CnAtf1_bZIP	125	227	159.9	140.9	212		CnAtf1_bZIP
	ScCst6_bZIP	CNAG_03976_bZIP	CnGsb1_bZIP	CaRca1_bZIP	CnHxl1_bZIP	CnAtf1_bZIP	

Fig. S8. *C. neoformans* Gsb1 bZIP domain. (a) Alignment of bZIP domains observed in Gsb1 and ATF/CREB, and AP-1-like homologs from several fungi and mammals. The DNA-binding

domain of the bZIP domain is boxed. (b) Phylogenic tree analysis of Gsb1-related bZIP proteins. (c) *C. neoformans* Gsb1 bZIP domain. Alignment of the bZIP domains observed in Gsb1 and ATF/CREB, and AP-1-like homologs from several fungi and mammals.

Supplementary Figure 9

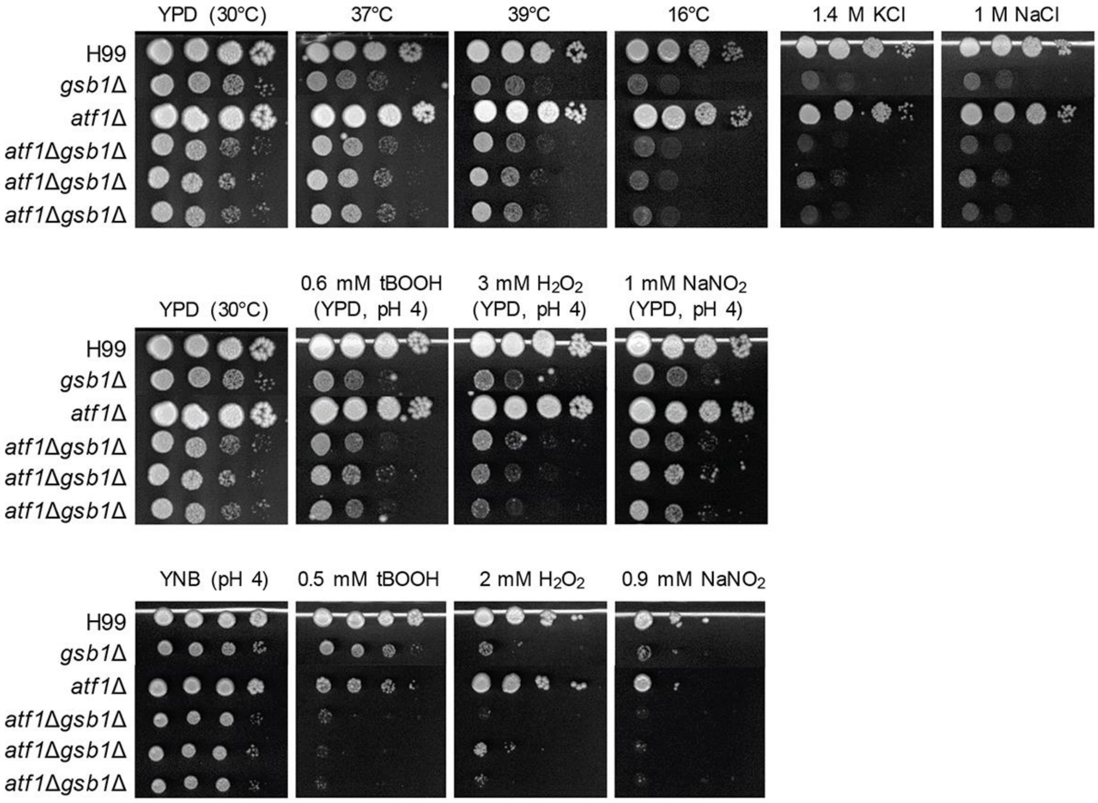

Fig. S9. Analysis of growth phenotype of the *atf1Δ gsb1Δ* double-deletion strains. Strains grown in YPD medium overnight were washed in sterile distilled water twice, resuspended to 10^7 cells/mL, diluted serially by 10-fold, and spotted onto solid YPD or YNB growth plates containing various stress reagents at indicated concentrations.

Supplementary Table S1. Strains used in this study

**Strain Name**	**Genotype**	**Parental strain**	**Reference**
H99	Serotype A *MAT*α		Perfect, et al.[5]
KN99	Serotype A *MAT***a**		Nielsen, et al.[6]
C06-586	*MAT*α CNAG_00871Δ::*NAT-STM#125**	H99	This study
C06-587	*MAT*α CNAG_00871Δ::*NAT-STM#125*	H99	This study
C06-592	*MAT*α CNAG_07940Δ::*NAT-STM#125*	H99	This study
C06-593	*MAT*α CNAG_07940Δ::*NAT-STM#125*	H99	This study
C06-595	*MAT*α *gsb1*Δ::*NAT-STM#125*	H99	This study
C06-596	*MAT*α *gsb1*Δ::*NAT-STM#125*	H99	This study
C06-597	*MAT*α *gsb1*Δ::*NAT-STM#125*	H99	This study
C06-599	*MAT*α *gsb1*Δ::*NAT-STM#125*	H99	This study
C08-712	*MAT***a** *gsb1*Δ::*NEO*	KN99	This study
C08-714	*MAT***a** *gsb1*Δ::*NEO*	KN99	This study
C07-672	*MAT*α *gsb1*Δ+*GSB1*	C06-599	This study
C07-685	*MAT*α *gsb1*Δ + *GSB1*-HA	C06-599	This study
C07-686	*MAT*α *gsb1*Δ + *GSB1*-HA	C06-599	This study
C08-733	*MAT*α *gsb1*Δ + *GSB1-GFP*	C06-599	This study
C08-703	*MAT*α *atf1*Δ *gsb1*Δ::*NEO*	YSB676	This study
C08-704	*MAT*α *atf1*Δ *gsb1*Δ::*NEO*	YSB676	This study
C08-705	*MAT*α *atf1*Δ *gsb1*Δ::*NEO*	YSB676	This study
YSB552	*MAT*α *ire1*Δ*::NAT-STM*#224	H99	Cheon, et al.[3]
YSB723	*MAT*α *hxl1*Δ*::NAT-STM#229*	H99	Cheon, et al.[3]
KK1	*MAT*α *cna1*Δ*::NAT-STM*#117	H99	Kojima, et al.[7]
KK3	*MAT*α *mpk1*Δ*::NAT-STM*#150	H99	Kojima, et al.[7]
YSB42	*MAT*α *cac1*Δ*::NAT-STM*#159	H99	Bahn, et al.[8]
YSB64	*MAT*α *hog1*Δ*::NAT-STM*#177	H99	Bahn, et al.[9]
YSB127	*MAT*α *cpk1*Δ*::NAT-STM*#184	H99	Bahn, et al.[10]
YSB676	*MAT*α *atf1*Δ::*NAT-STM*#220	H99	Kim, et al.[11]
YSB119	*MAT*α *aca1*Δ::*NAT-STM*#43 *ura5 ACA1-URA5*		Bahn, et al.[8]
YSB121	*MAT***a** *aca1*Δ::*NEO ura5 ACA1-URA5*		Bahn, et al.[8]

Supplementary Table S2. Plasmids and Primers used in this study

Plasmid	Description	Reference
pNAT-STM#125	NAT-resistant marker vector	Kim, et al.[12]
pJAF1	NEO-resistant marker vector	Fraser, et al.[13]
pJAFS1	NEO-resistant marker vector	Cheon, et al.[3]
pJAFS1-GSB1PT	NEO^r vector containing the promoter and terminator of *GSB1*	This study
pJAF1-gsb1DP1	NEO^r vector containing the *GSB1* promoter	This study
pJAF1-gsb1DP2	*gsb1*Δ::*NEO*^{r} disruption vector	This study
pJAFS1-GSB1R1	NEO^r vector containing the *GSB1* gene	This study
pJAFS1-GSB1R2NH	NEO^r vector containing the *GSB1* gene tagged with an N-terminal HA epitope	This study
pJAFS1-GSB1R3CH	NEO^r vector containing the *GSB1* gene tagged with a C-terminal HA epitope	This study
pJAFS1-GSB1R5NF	NEO^r vector containing the *GSB1* gene tagged with an N-terminal GFP epitope	This study
pJAFS1-GSB1R6CF	NEO^r vector containing the *GSB1* gene tagged with a C-terminal GFP epitope	This study
**Primer**	**Sequence (5' to 3')**	**Purpose**
M13Fe	GTAAAACGACGGCCAGTGAGC	Primer for disruption marker
B1455	AACTCCGTCGCGAGCCCCATCAAC	Double joint PCR primer
B1454	AAGGTGTTCCCCGACGACGAATCG	Double joint PCR primer
C814	CGCTAGAGAAGAGATGTAGAAAC	Primer for disruption marker
C790	ATTTCATTCCGGCCACCAATCG	Disruption primer for CNAG_00871
C791	GCTCACTGGCCGTCGTTTTACGGCTGTCCTCACCTGTTTGCTG	Disruption primer for CNAG_00871
C815	GTTTCTACATCTCTTCTCTAGCGGCTTCACTCTCATTCGCACCGG	Disruption primer for CNAG_00871
C793	AATCCCCCATACGGCTCAAGC	Disruption primer for CNAG_00871
C820	TATTGTCAGCTGGGTCTTGC	Disruption primer for CNAG_03976
C821	GCTCACTGGCCGTCGTTTTACTCCAGCGGATACAGATGATG	Disruption primer for CNAG_03976
C822	GTTTCTACATCTCTTCTCTAGCGGATTCCGAAGACTCTGACGA	Disruption primer for CNAG_03976
C823	CGTTTGATGGTAGGAAGGTG	Disruption primer for CNAG_03976
C824	GCCTTGCTTCCAAGACACTA	Disruption primer for CNAG_07560
C825	GCTCACTGGCCGTCGTTTTACGTCGGCTTGTCGAAGTATGT	Disruption primer for CNAG_07560
C826	GTTTCTACATCTCTTCTCTAGCGTTGAAACAGAGCCTCACAGC	Disruption primer for CNAG_07560
C827	GTCAGTGGCAGTTCCTTCAC	Disruption primer for CNAG_07560

C828	CCTGCTGTTATGTCATGGTG	Disruption primer for CNAG_07940
C829	GCTCACTGGCCGTCGTTTTACGAAG GTTAGCTCGCTGTTGA	Disruption primer for CNAG_07940
C830	GTTTCTACATCTCTTCTCTAGCGTTC TTATCACGCTCCTCTGG	Disruption primer for CNAG_07940
C831	AACGAAGTGACGGACATACG	Disruption primer for CNAG_07940
C505	TGTGGATGCTGGCGGAGGATA	Universal diagnostic primer
C522	GTCTCTGAAACCAGGAAG	Universal diagnostic primer
C842	GCACCACAACCACTGACCCTT	Diagnostic primer for CNAG_00871Δ
C843	TCTTCACACTGTTCCATCGCCT	Diagnostic primer for CNAG_00871Δ
C844	GGTCATCGTACCAGTCCATCGG	Diagnostic primer for CNAG_03976Δ
C845	CTGAAATTGCTCCTCCGTAC	Diagnostic primer for CNAG_03976Δ
C846	AGGGATACCGCCTCGGATCT	Diagnostic primer for CNAG_07560Δ
C847	ACGACGGACCCCATTGACAC	Diagnostic primer for CNAG_07560Δ
C848	GTGGAGGCGATGAGGAGAGAAC	Diagnostic primer for CNAG_07940Δ
C849	GCGTGAAAGACGAGGGCTTG	Diagnostic primer for CNAG_07940Δ
C904	CACGGTACCATTGAAGTTTCAGAGA GTTAGTGATAG	Cloning primer for the *GSB1* promoter
C905	GCGGCCGCTCTAGAGCTAGCTATTG TCAGTCGCGTCGATG	Cloning primer for the *GSB1* promoter
C906	GCTAGCTCTAGAGCGGCCGCTTTTT TGAAACAGAGCCTCACA	Cloning primer for the *GSB1* terminator
C907	GTCGTTAACTTTGATAGCGTTTTTT GTGATTG	Cloning primer for the *GSB1* terminator
C908	GCAAGCTAGCATGTCCGCGATCGAT TACC	Cloning primer for the *GSB1* ORF
C909	CAGGCGGCCGCCTATGAACTTCCTT CTTTTGCTTG	Cloning primer for the *GSB1* ORF
C910	GATGTTCCTGACTATGCGTCCGCGA TCGATTACCAC	Cloning primer for the *GSB1* ORF with an N-terminal HA tag
C519	GCAAGCTAGCATGTACCCATACGAT GTTCCTGACTATGCG	Cloning primer for the *GSB1* ORF with an N-terminal HA tag
C911	AGGAACATCGTATGGGTATGAACTT CCTTCTTTTGCTTGAG	Cloning primer for the *GSB1* ORF with a C-terminal HA tag
C912	CAGGCGGCCGCCTACGCATAGTCA GGAACATCGTATGGGTA	Cloning primer for the *GSB1* ORF with a C-terminal HA tag
C991	TGCTTGATATCAGAGGATGGATTG	Cloning primer for the *GSB1* disruption cassette
C992	CTTTCTAGAGGTACCGTCAGTGGCA GTTCCTTCAC	Cloning primer for the *GSB1* disruption cassette
C1065	GCAAGCTAGCATGGTGAGCAAGGG CGAG	Cloning primer for N-terminal GFP
C1066	CATGGATCCACCGCCACCCTTGTAC AGCTCGTCCATGCC	Cloning primer for N-terminal GFP

C1067	GGTGGCGGTGGATCCATGTCCGCG ATCGATTACC	Cloning primer for the *GSB1* gene tagged with an N-terminal GFP gene
C1068	GACCGCTGTGGATCTTCCAC	Cloning primer for the *GSB1* gene tagged with an N-terminal GFP gene
C1069	AGGTGCGGGAGTCGATAAAG	Cloning primer for the *GSB1* gene tagged with an N-terminal GFP gene
C1070	CATGGATCCACCGCCACCTGAACTT CCTTCTTTTGCTTG	Cloning primer for the *GSB1* gene tagged with an N-terminal GFP gene
C1071	GGTGGCGGTGGATCCATGGTGAGC AAGGGCGAG	Cloning primer for C-terminal GFP
C1072	CAGGCGGCCGCCTACTTGTACAGCT CGTCCATGC	Cloning primer for C-terminal GFP
C914	TGGAAACAAGGTTGTGAAAG	Diagnostic primer for complementation of *GSB1*
C919	ACCCAGAACGGAAGACAAGA	Diagnostic primer for complementation of *GSB1*
C846	AGGGATACCGCCTCGGATCT	Diagnostic primer for complementation of *GSB1*
C920	CGCGCAATTAACCCTCACTA	Diagnostic primer for complementation of *GSB1*
C816	AAAGCCATCAGCAACAACAG	qRT-PCR primer of CNAG_07560 (*GSB1*)
C817	GTGCTCTTTGCCTCTCCTTC	qRT-PCR primer of CNAG_07560 (*GSB1*)
C973	CCGCTAACATCATCCCTTCT	qRT-PCR primer of CNAG_06699 (*GAPDH*)
C974	CCACGACGGATACATCAGAG	qRT-PCR primer of CNAG_06699 (*GAPDH*)
C1013	AATTGAACGATTCTGCACCA	qRT-PCR primer of CNAG_00179 (*GPA2*)
C1014	TGTGGGCATGTAGTTTGGTT	qRT-PCR primer of CNAG_00179 (*GPA2*)
C1015	CCACCTTGTCCCTCTTGTTT	qRT-PCR primer of CNAG_02531 (*CPK1*)
C1016	CTCAAGTCTTGGGTTCGGAT	qRT-PCR primer of CNAG_02531 (*CPK1*)
C1017	CGTGAATTCTTGAGGAGCAA	qRT-PCR primer of CNAG_00125 (*CRG1*)
C1018	AGAGCTTTGCCTGTTTCGAT	qRT-PCR primer of CNAG_00125 (*CRG1*)
C1019	TCCTGTTCATATCGCTGCTC	qRT-PCR primer of CNAG_05866 (*PRM1*)
C1020	TTGGTGACCAGTGAGTTGGT	qRT-PCR primer of CNAG_05866 (*PRM1*)
C1021	TTTCACTCGCCATGTTCTTC	qRT-PCR primer of CNAG_01855 (*GPR2*)

C1022	AGGATCCAACGAGGATTGAG	qRT-PCR primer of CNAG_01855 (*GPR2*)
C1023	CTGTAATGGTTCCGACGATG	qRT-PCR primer of CNAG_03366 (*ZNF2*)
C1024	AGGATGGATGGCAAAGGTAG	qRT-PCR primer of CNAG_03366 (*ZNF2*)
C1025	TGTTCTTGGTGATGAACGGT	qRT-PCR primer of CNAG_06203 (*MAT2*)
C1026	CTCGCTGTATCATTCTCGGA	qRT-PCR primer of CNAG_06203 (*MAT2*)
C1146	CTAGGATGGGCTGTTATTCC	qRT-PCR primer of CNAG_09001 (NADH dehydrogenase subunit 1, ND1)
C1147	GCTGACCATCCAGCAAATAG	qRT-PCR primer of CNAG_09001 (NADH dehydrogenase subunit 1, ND1)
C1148	GCACCTAACATGCTAGGACAC	qRT-PCR primer of CNAG_09002 (Apocytochrome b)
C1149	CCTAGTAGCTTGTTAGGAATTGACCG	qRT-PCR primer of CNAG_09002 (Apocytochrome b)
C1150	CCATCACGACCACGTATGAC	qRT-PCR primer of CNAG_09003 (Small ribosomal protein subunit 3)
C1151	GGGAGTTGTTGATCGTTGGC	qRT-PCR primer of CNAG_09003 (Small ribosomal protein subunit 3)
C1152	GGAAACCGACGAGCAACAC	qRT-PCR primer of CNAG_09004 (Cytochrome C oxidase subunit 3, COIII)
C1153	CGTGAACCCCGTGGAATCC	qRT-PCR primer of CNAG_09004 (Cytochrome C oxidase subunit 3, COIII)
C1154	GGCTACCTCGAGCACATGCAG	qRT-PCR primer of CNAG_09005 (NADH:ubiquinone oxidoreductases 4, ND4)
C1155	GTTGCATCTGGCATCATACC	qRT-PCR primer of CNAG_09005 (NADH:ubiquinone oxidoreductases 4, ND4)
C1156	GGTACTGATTTCCTATCAACAG	qRT-PCR primer of CNAG_09006 (NADH:ubiquinone oxidoreductases 5, ND5)
C1157	CCTGCACCAAATAGACTTCC	qRT-PCR primer of CNAG_09006 (NADH:ubiquinone oxidoreductases 5, ND5)
C1158	CCAGCTGCTACAACTAATGC	qRT-PCR primer of CNAG_09007 (NADH dehydrogenase 6, ND6)
C1159	CAGCACCAGTATTAGCAAAAGCC	qRT-PCR primer of CNAG_09007 (NADH dehydrogenase 6, ND6)
C1160	GGACTATATACACACGGTCTGC	qRT-PCR primer of CNAG_09008 (ATP synthase subunit, ATP6)
C1161	CCAGCTGTCATATTTGCG	qRT-PCR primer of CNAG_09008 (ATP synthase subunit, ATP6)

C1162	CCTGATGCTTACGAAGGATGG	qRT-PCR primer of CNAG_09009 (Cytochrome c oxidase subunit 1, COI)
C1163	CCCCAGGGGTTCATAGATGC	qRT-PCR primer of CNAG_09009 (Cytochrome c oxidase subunit 1, COI)
C1164	GGTGCTTCATTCCTAGTCTC	qRT-PCR primer of CNAG_09010 (NADH dehydrogenase 2, ND2)
C1165	CTTTAGACCAGCTGCTGTAGC	qRT-PCR primer of CNAG_09010 (NADH dehydrogenase 2, ND2)
C1166	CCCTATTGCAGTAACTATGAG	qRT-PCR primer of CNAG_09011 (NADH dehydrogenase 3, ND3)
C1167	CTGAAATAGAAGATCGTTGGTCTG	qRT-PCR primer of CNAG_09011 (NADH dehydrogenase 3, ND3)
C1168	GGGGAATCAATCGAATTCGATAGC	qRT-PCR primer of CNAG_09012 (Cytochrome c oxidase subunit 2, COII)
C1169	GCCCCTGTTACTACAAATCG	qRT-PCR primer of CNAG_09012 (Cytochrome c oxidase subunit 2, COII)

*Each *NAT-STM#* indicates the Nat^r marker with a unique signature tag.

Supplementary Table S3. List of *C. neoformans* genes upregulated by more than two-fold under normal conditions

GO_Name	Gene	Feature ID	GO_Object Name	H99 (RPKM)	*gsb1*Δ (RPKM)	Ratio (*gsb1*Δ/H99)
Autophagy	CNAG_ 06892	CNAG_ 06892	Autophagy-related protein 3	38.849	89.786	2.311
Carbohydrate metabolic process	ATG26	CNAG_ 02834	Sterol 3-beta-glucosyltransferase	10.852	21.799	2.009
	CNAG_ 03247	CNAG_ 03247	Sterol 3beta-glucosyltransferase	20.141	45.361	2.252
	CNAG_ 05411	CNAG_ 05411	Endoglucanase	28.428	64.057	2.253
	CNAG_ 06931	CNAG_ 06931	Beta-glucosidase	4.372	19.973	4.569
	CNAG_ 00826	CNAG_ 00826	Dihydroxyacetone kinase	8.824	23.353	2.646
	CNAG_ 00057	CNAG_ 00057	Fructose-1,6-bisphosphatase I	169.410	350.967	2.072
	CNAG_ 00827	CNAG_ 00827	Ribose 5-phosphate isomerase	2.324	13.661	5.879
	SKN1	CNAG_ 00897	Glucosidase	64.466	148.040	2.296
	XFP1	CNAG_ 02230	Phosphoketolase	109.175	235.153	2.154
	CHI2	CNAG_ 03412	Chitinase	19.342	60.786	3.143
	CNAG_ 04025	CNAG_ 04025	Transaldolase	1.163	4.492	3.861
	CNAG_ 04631	CNAG_ 04631	Ribitol kinase	9.630	20.867	2.167
	CNAG_ 04744	CNAG_ 04744	Mannose-6-phosphate isomerase, class I	48.431	141.095	2.913
	CNAG_ 05458	CNAG_ 05458	Endo-1,3(4)-beta-glucanase	17.195	34.725	2.019
	GLH5	CNAG_ 05652	Cytoplasmic protein	85.759	176.955	2.063
	CNAG_ 06666	CNAG_ 06666	Alpha-1,4 glucan phosphorylase	3.744	20.031	5.350
	XFP2	CNAG_ 06923	Phosphoketolase	112.241	284.739	2.537
	CNAG_ 06936	CNAG_ 06936	Beta-glucosidase	4.174	8.527	2.043
	CNAG_ 07707	CNAG_ 07707	Glycoside hydrolase family 3 domain-containing protein	0.452	1.284	2.839
	GLH63	CNAG_ 02079	Cytoplasmic protein	85.603	178.741	2.088
	CNAG_ 06868	CNAG_ 06868	Phosphopyruvate hydratase	70.085	165.885	2.367
Cell cycle	CNAG_ 01037	CNAG_ 01037	DNA mismatch repair protein MLH3	13.250	27.256	2.057
	CNAG_ 07758	CNAG_ 07758	Uncharacterized protein	8.407	17.852	2.123
	CNAG_ 02658	CNAG_ 02658	Uncharacterized protein	29.577	63.177	2.136
	CNAG_ 05771	CNAG_ 05771	Serine/threonine-protein kinase TEL1	7.528	31.494	4.183
	ND4	CNAG_ 09005	NADH-ubiquinone oxidoreductase chain 4	0.152	0.323	2.129
	ND5	CNAG_ 09006	NADH-ubiquinone oxidoreductase chain 5	0.333	0.783	2.353
	ND2	CNAG_ 09010	NADH dehydrogenase subunit 2	0.037	0.296	8.068
	CNAG_ 00330	CNAG_ 00330	Transcription factor TFIIIB component b	14.379	28.906	2.010

Cellular nitrogen compound metabolic process	CNAG_ 02787	CNAG_ 02787	Dephospho-CoA kinase	45.936	93.425	2.034
	CNAG_ 02767	CNAG_ 02767	Transcription initiation factor TFIIE subunit alpha	24.155	48.835	2.022
	CNAG_ 02770	CNAG_ 02770	Transcription initiation factor TFIID subunit 11	43.403	88.641	2.042
	PMA1	CNAG_ 03565	Plasma membrane ATPase	55.089	147.223	2.672
	NCS6	CNAG_ 02122	Cytoplasmic tRNA 2-thiolation protein 1	26.982	58.922	2.184
	ATP6	CNAG_ 09008	ATP synthase subunit a	0.297	0.711	2.390
	CNAG_ 00027	CNAG_ 00027	Transcriptional activator	18.819	38.685	2.056
	CNAG_ 00505	CNAG_ 00505	Uncharacterized protein	9.418	21.050	2.235
	CLR3	CNAG_ 00871	Uncharacterized protein	2.242	5.106	2.278
	CNAG_ 00896	CNAG_ 00896	Transcription factor	6.841	15.443	2.257
	CNAG_ 02066	CNAG_ 02066	Uncharacterized protein	2.995	10.335	3.451
	CNAG_ 02525	CNAG_ 02525	Uncharacterized protein	2.880	5.935	2.060

Supplementary Table S4. List of upregulated or downregulated *C. neoformans* genes involved in the mating pathway and mating process under normal growth conditions

**Feature ID**	**GO Name/Function**	**Gene**	**H99 (RPKM)**	***gsb1Δ* (RPKM)**	**Ratio (*gsb1Δ*/H99)**
CNAG_ 00179	Signal transduction/heterotrimeric G protein alpha subunit B	*GPA2*	18.877	4.992	0.264
CNAG_ 02531	MAPK cascade/CMGC/MAPK/ERK protein kinase	*CPK1*	11.682	3.616	0.310
CNAG_ 00125	intracellular signal transduction/Crg1 (flbA. putative regulator of G protein, Crg1)	*CRG1*	7.335	3.022	0.412
CNAG_ 05866	plasma membrane fusion involved in cytogamy/conserved hypothetical protein pheromone-regulated multispanning membrane protein Prm1	*PRM1*	14.474	6.571	0.454
CNAG_ 01855	conserved hypothetical protein; G-coupled receptor protein	*GPR2*	48.098	25.130	0.522
CNAG_ 03366	conserved hypothetical protein: transcription factor	*ZNF2*	10.463	5.775	0.552
CNAG_ 07725	specific transcriptional repressor		4.381	9.606	2.193
CNAG_ 06203	conserved hypothetical protein; transcription factor (MAT2);	*MAT2*	3.315	9.891	2.984
CNAG_ 05970	protein phosphorylation/ STE/STE20/PAKA protein kinase	*PAK1*	6.256	34.800	5.563

Supplementary Table S5. Differentially regulated genes between WT and *gsb1*Δ under H_2O_2-induced oxidative stress condition

**Gene ID**	**Feature ID**	**DB_Object_Name**	**H99 (YPD)** RPKM	**H99 (H_2O_2)** RPKM	**Ratio [H99 (H_2O_2) /H99 (YPD)]**	***gsb1*Δ (YPD)** RPKM	***gsb1*Δ (H_2O_2)** RPKM	**Ratio [*gsb1*Δ (H_2O_2)/ *gsb1*Δ (YPD)]**
CNAG_ 00318	CNAG_ 00318	Actin-related protein 2/3 complex subunit 5	156.644	210.300	1.343	182.423	95.752	0.525
CNAG_ 02094	CNAG_ 02094	Arp2/3 complex 34 kDa subunit	136.520	216.750	1.588	132.614	123.202	0.929
CNAG_ 04196	CNAG_ 04196	Actin-binding protein	129.182	250.880	1.942	154.405	145.107	0.940
CNAG_ 04765	CNAG_ 04765	Actin-related protein 2/3 complex subunit 3	100.186	369.990	3.693	143.955	218.322	1.517
ARC40	CNAG_ 05878	Arp2/3 complex-mediated actin nucleation	176.993	348.940	1.971	208.410	255.341	1.225
WSP1	CNAG_ 02029	Wiskott–Aldrich syndrome protein	32.686	87.940	2.690	28.994	54.603	1.883
ACA1	CNAG_ 05218	Adenylyl cyclase-associated protein	96.745	175.100	1.810	94.927	117.183	1.234
CNAG_ 05734	CNAG_ 05734	Actin filament organization	34.615	80.285	2.319	42.016	44.400	1.057
CNAG_ 01918	CNAG_ 01918	Cytoskeletal regulatory protein binding protein	18.905	28.147	1.489	19.747	12.009	0.608
CAP2	CNAG_ 03900	Capping protein (Actin filament) muscle Z-line	60.154	101.910	1.694	73.811	65.454	0.887
CAP1	CNAG_ 03967	F-actin capping protein	78.715	263.350	3.346	76.707	153.723	2.004
CNAG_ 03864	CNAG_ 03864	Negative regulation of actin filament polymerization	63.261	107.530	1.700	70.062	79.598	1.136
CNAG_ 03843	CNAG_ 03843	NAK protein kinase, reorganization and function of the actin cytoskeleton	12.623	21.199	1.679	20.025	9.660	0.482
FKS1	CNAG_ 06508	1,3-β-glucan synthase component	87.998	128.040	1.455	90.151	66.487	0.738
CNAG_ 04033	CNAG_ 04033	α 1,3-glucosidase	37.499	74.668	1.991	38.035	81.396	2.140
CNAG_ 06931	CNAG_ 06931	β-glucosidase	4.372	5.399	1.235	19.973	8.761	0.439
CDA1	CNAG_ 05799	Chitin deacetylase	267.222	547.590	2.049	371.053	398.875	1.075
MP98 (CDA2)	CNAG_ 01230	Chitin deacetylase 2	330.033	401.350	1.216	445.130	476.326	1.070
CHS5	CNAG_ 05818	Chitin synthase	20.609	27.703	1.344	15.072	4.673	0.310
CHS6	CNAG_ 06487	Chitin synthase	17.878	18.518	1.036	25.849	2.203	0.085

CHS3	CNAG_ 05581	Chitin synthase 3	75.507	97.572	1.292	87.856	52.178	0.594
CHI2	CNAG_ 03412	Chitinase	19.342	57.572	2.976	60.786	36.739	0.604
CNAG_ 05411	CNAG_ 05411	Endoglucanase	28.428	337.310	11.865	64.057	142.151	2.219
CNAG_ 00939	CNAG_ 00939	Glucan 1,3-β-glucosidase	1.695	1.121	0.661	0.820	5.047	6.158
CNAG_ 06336	CNAG_ 06336	Glucan 1,3-β-glucosidase	23.417	81.029	3.460	43.554	39.335	0.903
EXG104	CNAG_ 02225	Glucan 1,3-β-glucosidase	2.701	26.326	9.747	3.908	6.622	1.694
KRE6	CNAG_ 00914	Glucosidase	151.712	131.940	0.870	152.494	110.440	0.724
SKN1	CNAG_ 00897	Glucosidase	64.466	79.546	1.234	148.040	36.073	0.244
KRE61	CNAG_ 06835	Glucosidase	10.310	15.184	1.473	11.159	9.877	0.885
CNAG_ 01221	CNAG_ 01221	Cis-prenyltransferase	39.899	139.830	3.505	39.501	55.769	1.412
CNAG_ 04364	CNAG_ 04364	Oligosaccharyl transferase stt3 subunit	78.275	94.994	1.214	69.229	42.981	0.621
OST1	CNAG_ 04715	Oligosaccharyltransferase, α subunit	67.844	143.760	2.119	55.525	46.912	0.845
OST2	CNAG_ 00473	Oligosaccharyltransferase, ε subunit	107.875	190.790	1.769	129.907	45.128	0.347
WBP1	CNAG_ 04743	Oligosaccharyltransferase, β subunit	120.501	65.869	0.547	83.702	25.769	0.308
CNAG_ 02855	CNAG_ 02855	Oligosaccharyltransferase, δ subunit	93.396	90.987	0.974	122.340	63.624	0.520
ALG2	CNAG_ 00926	Glycolipid mannosyltransferase	38.211	55.303	1.447	37.990	16.806	0.442
OCH1	CNAG_ 00744	α-1,6-mannosyltransferase	36.574	93.781	2.564	47.325	35.248	0.745
MNN2	CNAG_ 06782	α-1,2-mannosyltransferase	51.471	586.360	11.392	74.750	222.453	2.976
HOC1	CNAG_ 05836	α-1,6-mannosyltransferase	60.447	60.824	1.006	55.710	24.869	0.446
HOC2	CNAG_ 01214	α-1,6-mannosyltransferase	8.976	24.790	2.762	14.275	33.151	2.322
HOC3	CNAG_ 00158	α-1,6-mannosyltransferase	27.491	7.588	0.276	36.097	4.329	0.120
CNAG_ 01050	CNAG_ 01050	Galactosyltransferases	25.400	27.753	1.093	29.137	13.879	0.476
CANG_ 06918	CNAG_ 06918	Galactosyltransferases	9.913	69.416	7.002	18.373	23.244	1.265
CNAG_ 01385	CNAG_ 01385	Galactosyltransferases	37.073	190.690	5.144	50.790	74.505	1.467
PMT1	CNAG_ 06834	Protein O-mannosyl transferase	58.938	206.230	3.499	51.745	55.977	1.082
PMT2	CNAG_ 04763	Protein O-mannosyl transferase	54.819	121.290	2.213	47.677	50.141	1.052
PMT4	CNAG_ 00996	Protein O-mannosyl transferase	62.137	47.794	0.769	51.980	52.429	1.009

KRT3	CANG_ 03832	α-1,2-mannosyltransferase	68.928	83.868	1.217	83.459	45.812	0.549
CNAG_ 00873	CNAG_ 00873		91.266	683.490	7.489	180.223	151.224	0.839
CNAG_ 00482	CNAG_ 00482	26S proteasome regulatory subunit N10	187.006	416.700	2.228	174.559	189.271	1.084
DOA4	CNAG_ 00757	Ubiquitin carboxyl-terminal hydrolase 8	16.698	45.311	2.714	20.532	30.104	1.466
UFD4	CNAG_ 01251	E3 ubiquitin-protein ligase TRIP12	35.293	58.141	1.647	52.954	49.127	0.928
CNAG_ 01732	CNAG_ 01732	Nuclear protein localization protein 4	64.824	106.550	1.644	59.716	73.538	1.231
CUL3	CNAG_ 03138	Ubiquitin-protein ligase	22.087	36.736	1.663	21.091	24.003	1.138
CNAG_ 04958	CNAG_ 04958	Ubiquitin fusion degradation protein 1	68.464	126.450	1.847	70.810	85.351	1.205
CNAG_ 05195	CNAG_ 05195	Ubiquitin-conjugation factor E4 B	36.524	78.985	2.163	42.883	68.481	1.597
CNAG_ 07333	CNAG_ 07333	Ubiquitin carboxyl-terminal hydrolase 25/28	31.572	43.935	1.392	32.900	22.151	0.673
UBP16	CNAG_ 00187	Ubiquitin carboxyl-terminal hydrolase 1	48.233	43.330	0.898	55.868	31.497	0.564
YUH1	CNAG_ 00180	Ubiquitin carboxyl-terminal hydrolase	75.945	218.480	2.877	83.086	154.849	1.864
RPN12	CNAG_ 03721	26S proteasome regulatory subunit N12	176.765	290.340	1.643	241.496	203.875	0.844
NEP1	CNAG_ 03935	Endothelin-converting enzyme	44.395	134.220	3.023	53.467	96.275	1.801
CNAG_ 04275	CNAG_ 04275	Metalloendopeptidase	43.197	92.869	2.150	34.144	72.591	2.126
CNAG_ 04380	CNAG_ 04380	Peptidase	26.788	55.501	2.072	51.815	16.000	0.309
CNAG_ 04502	CNAG_ 04502	Peptide hydrolase	40.084	55.850	1.393	45.180	49.122	1.087
CNAG_ 04524	CNAG_ 04524	Zinc metalloprotease	20.770	314.320	15.134	43.286	94.530	2.184
CNAG_ 05446	CNAG_ 05446	Kexin	30.497	49.454	1.622	46.080	27.241	0.591
RPT5	CNAG_ 06153	26S protease regulatory subunit 6A-B	207.354	281.240	1.356	208.394	253.249	1.215
CNAG_ 07771	CNAG_ 07771	Peptidase	42.685	103.300	2.420	45.734	79.229	1.732
CNAG_ 02562	CNAG_ 02562	Acyl-CoA dehydrogenase	9.209	11.932	1.296	17.257	5.728	0.332
ACD3	CNAG_ 03666	Acyl-CoA dehydrogenase, fatty acid synthesis	9.790	24.162	2.468	7.297	6.654	0.912
CNAG_ 06783	CNAG_ 06783	Enoyl reductase, fatty acid synthesis	7.023	10.571	1.505	10.432	4.590	0.440
CANG_ 00403	CNAG_ 00403	γ-butyrobetaine,2-oxoglutarate dioxygenase	26.266	31.959	1.217	23.041	10.718	0.465

CNAG_ 03870	CNAG_ 03870	Δ8-fatty-acid desaturase	29.629	39.450	1.331	40.550	41.594	1.026
FRE2	CNAG_ 06821	Ferric reductase transmembrane component 4	41.880	44.243	1.056	34.660	16.245	0.469
CNAG_ 05777	CNAG_ 05777	L-ascorbic acid binding	26.303	32.476	1.235	63.439	25.233	0.398
CNAG_ 05346	CNAG_ 05346	Sarcosine oxidase, glycine synthesis	5.297	7.931	1.497	3.450	1.634	0.474
ADH50	CNAG_ 00515	Mannitol dehydrogenase	5.252	34.839	6.633	10.726	5.411	0.504
PYX3	CNAG_ 02592	Thioredoxin reductase GliT	2.754	8.684	3.154	6.078	3.433	0.565
CNAG_ 01540	CNAG_ 01540	Dehydrogenase	10.762	14.762	1.372	13.177	7.874	0.598
CNAG_ 06529	CNAG_ 06529	Oxidoreductase activity	17.635	58.299	3.306	30.952	16.238	0.525
SER30	CNAG_ 04085	Oxidoreductase	9.497	13.495	1.421	12.064	6.343	0.526
SER33	CNAG_ 04955	Oxidoreductase	30.454	57.629	1.892	36.255	35.436	0.977
CNAG_ 03050	CNAG_ 03050	Amine oxidase	3.138	8.760	2.792	5.221	3.767	0.722
CNAG_ 06524	CNAG_ 06524	Ferric reductase	0.778	1.841	2.364	1.940	1.283	0.661
LOT6	CNAG_ 03185	Low temperature-responsive protein	22.882	46.939	2.051	48.335	35.148	0.727
CNAG_ 01080	CNAG_ 01080	Glycolate oxidase, FMN binding	4.665	7.697	1.650	4.354	1.635	0.376
CNAG_ 02758	CNAG_ 02758	NADH:flavin oxidoreductase/NAD H oxidase	16.657	24.196	1.453	30.006	25.331	0.844
RNR2	CNAG_ 01915	Ribonucleoside-diphosphate reductase subunit M2	274.593	713.450	2.598	214.297	156.301	0.729
CNAG_ 00692	CNAG_ 00692	FAD dependent oxidoreductase	20.520	40.990	1.998	33.275	24.785	0.745
CNAG_ 06785	CNAG_ 06785	Kynurenine 3-monooxygenase, FAD binding	11.162	14.339	1.285	13.454	6.307	0.469
CNAG_ 03556	CNAG_ 03556	Dimethylaniline monooxygenase, FAD binding	12.710	17.221	1.355	16.582	16.477	0.994
FOR2	CNAG_ 00542	Salicylate hydroxylase, FAD binding	3.920	6.516	1.662	3.216	2.457	0.764
CNAG_ 00735	CNAG_ 00735	Aldehyde dehydrogenase	27.940	42.752	1.530	30.904	8.128	0.263
CNAG_ 02377	CNAG_ 02377	Aldehyde dehydrogenase	52.112	69.567	1.335	52.942	43.740	0.826
CNAG_ 06977	CNAG_ 06977	L-iditol 2-dehydrogenase	4.777	11.546	2.417	3.741	3.190	0.853
CNAG_ 01075	CNAG_ 01075	Methylmalonate-semialdehyde dehydrogenase	1.266	5.580	4.407	0.575	0.525	0.912
UOX1	CNAG_ 04307	Uricase, peroxisomal	32.455	64.559	1.989	28.367	26.450	0.932

TFD2	CNAG_ 01542	Taurine catabolism dioxygenase TauD	2.790	27.219	9.754	4.887	4.793	0.981
CNAG_ 06249	CNAG_ 06249	Taurine catabolism dioxygenase TauD	8.609	24.908	2.893	12.913	10.249	0.794
CNAG_ 01220	CNAG_ 01220	Sulfhydryl oxidase	48.786	75.209	1.542	75.606	86.248	1.141
CNAG_ 01569	CNAG_ 01569	Protein import into mitochondrial matrix	30.412	54.279	1.785	33.909	28.839	0.850
ZTA11	CNAG_ 02933	Quinone oxidoreductase	21.852	42.015	1.923	56.175	54.368	0.968
CNAG_ 02979	CNAG_ 02979	Mitochondrial matrix	10.961	17.040	1.555	22.576	24.637	1.091
ACO1	CNAG_ 03427	Aconitate hydratase, mitochondrial	8.933	12.949	1.450	7.889	7.053	0.894
CNAG_ 07363	CNAG_ 07363	Isocitrate dehydrogenase [NAD] subunit	270.965	627.310	2.315	262.610	472.895	1.801
IDH1	CNAG_ 07851	Isocitrate dehydrogenase, NAD-dependent	334.823	695.720	2.078	344.048	599.932	1.744
CNAG_ 09002	CNAG_ 09002	Cytochrome b	0.399	1.506	3.770	0.955	1.213	1.271
ND4	CNAG_ 09005	NADH-ubiquinone oxidoreductase chain 4	0.152	1.264	8.326	0.323	0.663	2.051
ND5	CNAG_ 09006	NADH-ubiquinone oxidoreductase chain 5	0.333	2.525	7.593	0.783	1.768	2.259
ATP6	CNAG_ 09008	ATP synthase subunit a	0.297	1.703	5.725	0.711	0.753	1.059
COI	CNAG_ 09009	Cytochrome c oxidase subunit 1	1.129	3.318	2.939	2.818	3.508	1.245
COII	CNAG_ 09012	Cytochrome c oxidase subunit 2	0.620	3.070	4.953	0.850	1.717	2.020
ND3	CNAG_ 09011	NADH-ubiquinone oxidoreductase chain 3	0.217	0.273	1.257	0.324	0.110	0.339
CCP1	CNAG_ 01138	Cytochrome c peroxidase, mitochondrial	144.127	297.900	2.067	97.976	78.302	0.799
CNAG_ 00839	CNAG_ 00839	Mitochondrial protein	8.422	11.190	1.329	9.710	11.198	1.153
GPA1	CNAG_ 04505	Guanine nucleotide-binding protein subunit alpha	50.455	38.398	0.761	62.764	26.395	0.421
CAC1	CNAG_ 03202	Adenylate cyclase	12.636	10.956	0.867	25.749	12.448	0.483
PDE2	CNAG_ 07470	Phosphodiesterase	27.500	21.758	0.791	39.149	19.450	0.497
CNAG_ 02511	CNAG_ 02511	Mitogen-activated protein kinase	17.023	18.467	1.085	24.729	12.700	0.514
PAK1	CNAG_ 05970	STE/STE20/PAKA protein kinase	6.256	9.989	1.597	34.800	10.063	0.289
CNAG_ 02381	CNAG_ 02381	Regulation of Rho protein signal transduction	0.383	1.010	2.639	1.064	0.611	0.574

CNAG_ 02398	CNAG_ 02398	Regulation of Rho protein signal transduction	50.890	73.833	1.451	57.194	46.830	0.819
ROM21	CNAG_ 03680	Regulation of Rho protein signal transduction	15.425	10.912	0.707	12.375	4.848	0.392
CNAG_ 02458	CNAG_ 02458	GTPase activating protein	33.629	51.172	1.522	34.413	14.625	0.425
CNAG_ 01982	CNAG_ 01982	Uncharacterized protein	10.136	21.360	2.107	20.257	10.106	0.499
CNAG_ 03248	CNAG_ 03248	Rho GTPase activator	42.233	63.752	1.510	70.751	43.701	0.618
LRG1	CNAG_ 05703	Rho GTPase activator	28.098	22.812	0.812	40.250	17.652	0.439
ROM2	CNAG_ 04119	Rho guanyl-nucleotide exchange factor	24.641	19.160	0.778	24.874	8.248	0.332
PKC1	CNAG_ 01845	Protein kinase C	71.954	74.584	1.037	79.016	27.868	0.353
BCK1	CNAG_ 04755	STE/STE11/BCK1 protein kinase	32.617	57.045	1.749	60.731	36.511	0.601
MPK1	CNAG_ 04514	Mitogen-activated protein kinase	58.372	45.021	0.771	77.726	24.315	0.313
KIC1	CNAG_ 00405	STE/STE20/YSK protein kinase	15.344	29.404	1.916	20.875	14.651	0.702
CNAG_ 03893	CNAG_ 03893	MAP kinase phosphatase	9.262	28.335	3.059	13.668	9.271	0.678
CNAG_ 04335	CNAG_ 04335	Phosphatidylinositol 4-kinase	15.514	22.650	1.460	19.223	13.969	0.727
CNAG_ 03821	CNAG_ 03821	Phosphatidylinositol 3-kinase	17.203	29.627	1.722	26.355	11.129	0.422
CNAG_ 03024	CNAG_ 03024	AGC protein kinase	10.827	15.639	1.444	26.810	25.988	0.969
SKN7	CNAG_ 03409	Osomolarity two-component system, response regulator SKN7	27.723	29.502	1.064	30.708	14.905	0.485
CNAG_ 03876	CNAG_ 03876	Ras family protein	123.590	110.850	0.897	139.127	75.772	0.545
CNAG_ 01533	CNAG_ 01533	Rho GTPase activator	21.995	18.349	0.834	20.443	6.679	0.327
CNAG_ 04771	CNAG_ 04771	Rab family protein	95.528	159.290	1.667	109.666	95.405	0.870
CNAG_ 02116	CNAG_ 02116	Arf/Sar family protein	19.629	16.404	0.836	26.731	8.007	0.300
CNAG_ 05068	CNAG_ 05068	GTP-binding protein ypt1	373.002	728.380	1.953	461.317	524.805	1.138
CNAG_ 00625	CNAG_ 00625	ARF guanyl-nucleotide exchange factor	18.118	27.048	1.493	23.202	13.026	0.561
CNAG_ 06049	CNAG_ 06049	GTP-binding protein ryh1	79.727	59.469	0.746	105.425	53.860	0.511
SCP1	CNAG_ 01580	SREBP signaling pathway	23.763	22.456	0.945	25.026	13.612	0.544
CNAG_ 03583	CNAG_ 03583	Histone acetyltransferase (MYST family)	17.800	32.815	1.844	16.951	5.987	0.353

CLR3	CNAG_00871	conserved hypothetical protein (bZIP), capsule-linked regulator	2.242	4.018	1.792	5.106	2.735	0.536
CNAG_05990	CNAG_05990	Uncharacterized protein	6.879	10.105	1.469	6.055	1.506	0.249
CNAG_05333	CNAG_05333	Uncharacterized protein	1.220	1.995	1.635	1.990	1.094	0.550
CNAG_02066	CNAG_02066	Putative transcription factor HALR/MLL3, involved in embryonic development	2.995	5.660	1.890	10.335	6.841	0.662
YRM103	CNAG_04093	Uncharacterized protein	1.117	2.428	2.173	1.496	1.078	0.720
CNAG_02603	CNAG_02603	Zn-finger, early growth response protein 1	2.616	5.619	2.148	3.230	2.392	0.741
CNAG_05066	CNAG_05066	Zn-finger	2.827	16.548	5.854	8.335	6.554	0.786
BWC2	CNAG_02435	GATA-4/5/6 transcription factors, white collar 2, involved in the photosensory response	134.162	260.160	1.939	151.811	122.733	0.808
CNAG_05019	CNAG_05019	Uncharacterized protein	11.315	22.749	2.010	10.130	8.342	0.824
CNAG_01847	CNAG_01847	Putative transcription factor HALR/MLL3, involved in embryonic development	19.156	25.841	1.349	14.272	12.839	0.900
CNAG_04036	CNAG_04036	Heat shock transcription factor	6.707	13.564	2.022	5.356	4.933	0.921
CNAG_02695	CNAG_02695	Nuclear actin-related protein involved in chromatin remodeling	26.907	61.832	2.298	55.813	52.465	0.940
CNAG_04023	CNAG_04023	Transcription factor CHX10 and related HOX domain proteins	20.489	28.608	1.396	23.820	23.851	1.001
ECM2201	CNAG_00883	Transcription factor, involved in the normal capsular growth	11.042	22.645	2.051	15.840	16.130	1.018
CNAG_05380	CNAG_05380	Uncharacterized protein	34.425	48.435	1.407	42.002	48.391	1.152
CNAG_00055	CNAG_00055	Uncharacterized protein	48.763	183.040	3.754	51.396	59.961	1.167
HAP2	CNAG_07435	CCAAT-binding factor, subunit B (HAP2), involved in the mitochondrial function in fungi	19.799	78.607	3.970	21.051	24.819	1.179
CNAG_04284	CNAG_04284	Uncharacterized protein	189.048	289.790	1.533	193.425	233.627	1.208
CNAG_05255	CNAG_05255	Uncharacterized protein	24.165	34.682	1.435	33.453	45.227	1.352
ASG1	CNAG_03849	member of the Gal4p family of zinc cluster proteins	22.661	44.066	1.945	25.283	35.353	1.398

RLM1	CNAG_ 03998	MADS box transcription factor, regulator of the cell wall integrity pathway	23.982	80.414	3.353	29.532	43.126	1.460
CNAG_ 00559	CNAG_ 00559	Uncharacterized protein	85.772	215.650	2.514	159.665	234.697	1.470
CNAG_ 02305	CNAG_ 02305	Predicted DNA damage inducible protein	9.003	30.547	3.393	10.774	16.601	1.541
CNAG_ 04041	CNAG	Uncharacterized protein	17.305	33.124	1.914	25.174	39.106	1.553
CNAG_ 03768	CNAG_ 03768	Nuclear receptor coregulator SMRT/SMRTER, contains Myb-like domains	18.374	37.194	2.024	16.724	25.988	1.554
CNAG_ 06691	CNAG_ 06691	Tuftelin-interacting protein TIP39, contains G-patch domain	32.034	96.687	3.018	45.308	72.433	1.599
CNAG_ 02428	CNAG_ 02428	GC-rich sequence DNA-binding factor	35.304	68.942	1.953	42.532	68.071	1.600
CNAG_ 04895	CNAG_ 04895	Calcium-responsive transcription coactivator	22.721	76.355	3.361	42.403	68.106	1.606
CNAG_ 05642	CNAG_ 05642	Ca^{2+}-modulated nonselective cation channel polycystin	21.519	43.336	2.014	31.111	53.314	1.714
GAT201	CNAG_ 01551	GATA-4/5/6 transcription factors, involved in the capsule regulation	15.581	59.024	3.788	17.424	30.433	1.747
GAT204	CNAG_ 06762	GATA-4/5/6 transcription factors, in the capsule-independent anti-phagocytic mechanism	24.183	89.133	3.686	37.252	67.140	1.802
CNAG_ 06156	CNAG_ 06156	Uncharacterized protein	10.420	33.384	3.204	20.208	35.879	1.776
CNAG_ 06150	CNAG_ 06150	Chaperone regulator	750.969	204.600	0.272	472.017	1575.156	3.337
CNAG_ 03944	CNAG_ 03944	Cyclophilin	330.533	107.320	0.325	258.857	783.241	3.026
FRR1	CNAG_ 03682	Peptidyl-prolyl cis-trans isomerase-like 4	977.727	518.400	0.530	682.646	1655.373	2.425
HSP10	CNAG_ 03892	Chaperonin-containing T-complex protein 1 subunit β	890.746	140.490	0.158	369.168	1035.223	2.804
CNAG_ 01696	CNAG_ 01696	Chaperonin-containing T-complex protein 1 subunit ε	110.421	41.965	0.380	120.611	563.439	4.672
HSP60	CNAG_ 03891	Chaperonin-containing T-complex protein 1 subunit δ	701.509	29.046	0.041	321.879	254.291	0.790

CNAG_ 00447	CNAG_ 00447	Chaperonin-containing T-complex protein 1 subunit γ	269.435	29.928	0.111	232.332	211.391	0.910
CNAG_ 00058	CNAG_ 00058	Chaperonin-containing T-complex protein 1 subunit θ	211.138	29.527	0.140	199.211	166.948	0.838
CNAG_ 02736	CNAG_ 02736	Calnexin	186.323	16.137	0.087	243.837	229.694	0.942
CNAG_ 02440	CNAG_ 02440	Cation-transporting ATPase	67.656	5.635	0.083	46.649	47.477	1.018
CNAG_ 05932	CNAG_ 05932	Peptidyl-prolyl cis-trans isomerase	322.892	41.681	0.129	209.728	334.613	1.595
CNAG_ 07346	CNAG_ 07346	Peptidyl-prolyl cis-trans isomerase	225.179	42.573	0.189	141.781	133.627	0.942
CNAG_ 03459	CNAG_ 03459	Peptidyl-prolyl cis-trans isomerase	239.865	34.366	0.143	203.708	203.835	1.001
CNAG_ 04304	CNAG_ 04304	Peptidyl-prolyl cis-trans isomerase	285.155	46.724	0.164	229.306	233.699	1.019
CNAG_ 01568	CNAG_ 01568	Prefolding α subunit	217.566	34.913	0.160	175.708	181.887	1.035
CNAG_ 07860	CNAG_ 07860	Peptidyl-prolyl cis-trans isomerase-like 2	21.090	73.863	3.502	34.238	40.900	1.195
CNAG_ 04171	CNAG_ 04171	Molecular chaperon ROT1	130.113	333.470	2.563	169.307	151.859	0.897
CNAG_ 03627	CNAG_ 03627	Hsp60-like protein	1645.832	928.560	0.564	1393.317	679.826	0.488
CNAG_ 03486	CNAG_ 03486	Chaperonin-containing T-complex protein 1 subunit ζ	174.528	80.822	0.463	90.177	33.946	0.376
CNAG_ 04805	CNAG_ 04805	Chaperone regulator	66.609	24.094	0.362	79.858	32.460	0.406
CPR3	CNAG_ 01636	Chaperonin GroES	108.671	36.155	0.333	109.602	48.084	0.439
CNAG_ 02825	CNAG_ 02825	Argininosuccinate lyase	167.354	49.051	0.293	146.965	348.352	2.370
ARG3	CNAG_ 02812	Ornithine carbamoyltransferase	191.819	195.890	1.021	174.288	578.350	3.318
CNAG_ 01238	CNAG_ 01238	Arginine biosynthesis bifunctional protein ArgJ, mitochondrial	79.653	7.598	0.095	108.617	98.706	0.909
CNAG_ 02418	CNAG_ 02418	Asparagine-tRNA ligase	293.846	211.470	0.720	136.875	584.670	4.272
CNAG_ 07400	CNAG_ 07400	Aspartate-tRNA(Asn) ligase	271.290	41.326	0.152	181.317	241.074	1.330
CNAG_ 03701	CNAG_ 03701	Pentafunctional AROM polypeptide	67.627	18.121	0.268	52.082	86.655	1.664
CNAG_ 05623	CNAG_ 05623	Chorismate synthase (ARO2)	226.456	26.603	0.117	222.136	281.004	1.265
CNAG_ 05028	CNAG_ 05028	Cysteine synthase	153.578	18.041	0.117	121.748	185.835	1.526
CNAG_ 07563	CNAG_ 07563	Glutamate-tRNA ligase	29.920	6.571	0.220	38.885	60.203	1.548
CNAG_ 05900	CNAG_ 05900	Glycine-tRNA ligase	292.802	23.638	0.081	231.554	200.898	0.868
HIS7	CNAG_ 00743	Imidazoleglycerol phosphate synthase, cyclase subunit	109.195	58.745	0.538	110.695	287.516	2.597

HIS5	CNAG_ 03812	Histidinol-phosphate transaminase	142.731	119.910	0.840	163.702	809.947	4.948
SER3	CNAG_ 00774	Phosphoglycerate dehydrogenase	174.671	26.303	0.151	133.778	540.429	4.040
LYS21	CNAG_ 00992	Homocitrate synthase, mitochondrial	373.811	310.640	0.831	401.474	1683.454	4.193
MET3	CNAG_ 04215	Sulfate adenylyltransferase	314.078	20.522	0.065	165.419	261.378	1.580
CNAG_ 02270	CNAG_ 02270	Homoserine O-acetyltransferase	29.367	16.407	0.559	26.382	169.856	6.438
CNAG_ 05122	CNAG_ 05122	Homoserine O-acetyltransferase	64.118	53.637	0.837	83.026	194.205	2.339
MET5	CNAG_ 05070	Sulfite reductase (NADPH) hemoprotein, beta-component	152.067	5.294	0.035	85.224	63.362	0.743
CNAG_ 02686	CNAG_ 02686	Cystathionine beta-lyase	58.592	42.653	0.728	84.855	257.456	3.034
CNAG_ 01305	CNAG_ 01305	Methionine-tRNA ligase	179.526	51.248	0.285	182.914	249.985	1.367
CNAG_ 04776	CNAG_ 04776	Homoserine dehydrogenase (HOM6)	169.418	16.425	0.097	177.742	169.466	0.953
ILV1	CNAG_ 07529	Threonine dehydratase	56.444	22.589	0.400	82.206	21.622	0.263
DFR1	CNAG_ 04764	Dihydrofolate reductase	60.516	47.087	0.778	66.743	31.975	0.479
CNAG_ 02818	CNAG_ 02818	Aminomethyltransferase	51.569	28.626	0.555	82.314	17.554	0.213
CNAG_ 06316	CNAG_ 06316	Glycine cleavage system H protein	109.019	58.711	0.539	144.537	46.214	0.320
CNAG_ 02366	CNAG_ 02366	4-aminobutyrate aminotransferase (UGA1)	32.594	49.588	1.521	31.432	19.181	0.610
CNAG_ 04344	CNAG_ 04344	Galactonate dehydratase	8.392	12.598	1.501	10.693	4.355	0.407

Supplementary Table S6. Putative *C. neoformans* bZIP proteins

H99 ID	Amino acids	Domain analysis (by Prosite)[1]		Gene Name (Cn (H99) Database)[2]	Gene name	Reference	Reported functions in *C. neoformans*
CNAG _07560	403 aa	BZIP	124-173	Conserved hypothetical protein	*GSB1*	General Stress-related bZIP protein; In this study	Involved in cellular responses to diverse stresses, particularly to oxidative stress, in cell mating and virulence
CNAG _00871	499 aa	BZIP	64 - 102	Conserved hypothetical protein	*CLR3*	FungiDB reserved gene name; Jung, et al.[14]; In this study	Deletion generated no apparent sensitivity to various stresses.
CNAG _03976	508 aa	BZIP	269-332	Conserved hypothetical protein			
CNAG _07940	342 aa	BZIP	87-134	Conserved hypothetical protein	*BZP5*	Basic region leucine zipper 5; Jung, et al.[14]; In this study	Deletion generated no apparent sensitivity to various stresses.
CNAG _06134	407 aa	BZIP	62-107	Conserved hypothetical protein	*BZP1 (HXL1)*	Basic region leucine zipper 1 (HAC1 and XBP1-Like gene 1); Cheon, et al.[3]	A downstream factor of Ire1 in UPR; involved in ER stress, cell-wall stress, thermotolerance, and antifungal drug resistance; essential for virulence
CNAG _00055	388 aa	BZIP_ BASIC	255-269	Conserved hypothetical protein			
CNAG _00132	715 aa	BZIP_ BASIC	647-662	Conserved hypothetical protein			
CNAG _00239	701 aa	BZIP_ BASIC	161-176	Conserved hypothetical protein	*YAP1 (BAP1)*	Yeast AP-1 like protein 1; Paul, et al.[15], Brown, et al.[16]	Associated with diverse oxidative stress agents and azole drugs but not involved in virulence
CNAG _00559	301 aa	BZIP	233-293	Conserved hypothetical protein	*BZP3*	Basic region leucine zipper 3; Jung, et al.[14]	Associated with susceptibility to fluconazole, amphotericin B, 5-flucyotosine, and fludioxonil
CNAG _00732	303 aa	BZIP_ BASIC	7-20	Conserved hypothetical protein			
CNAG _01242	718 aa	BZIP_ BASIC	139 - 154	Conserved hypothetical protein	*HAPX*	HapX; Jung, et al.[17]	A regulator for iron homeostasis; Loss of HapX resulted in a modest virulence defect.
CNAG _02788	405 aa	No hit		Conserved hypothetical protein			

CNAG _03125	652 aa	No hit		Conserved hypothetical protein			
CNAG _03346	408 aa	BZIP	317-375	Conserved hypothetical protein	*BZP4*	Basic region leucine zipper 4; Jung, et al.[14]	Deletion mutants showed increased resistance to amphotericin B.
CNAG _04090	618 aa	BZIP	539-602	Activating transcription factor 2	*ATF1*	Activating Transcription Factor 1; Missall and Lodge[18]	Required for induction of thioredoxin genes under oxidative stress condition and resistance to tertbutylhydroperoxide
CNAG _04263	776 aa	BZIP GATA _ZN_FINGER_2	66-111 622-680	Conserved hypothetical protein	*BZP2*	Jung, et al.[14]	Deletion mutants showed increased susceptibility to antifungal agents.
CNAG _04630	689 aa	BZIP_ BASIC	331-346	Conserved hypothetical protein	*YAP2*	Jung, et al.[14]	Deletion mutants showed increased susceptibility to fluconazole, amphotericin B, 5-flucyotosine, and fludioxonil.
CNAG _04798	291 aa	BZIP	190-247	Regulatory protein cys-3		Regulatory protein cys-3	
CNAG _04908	453 aa			Conserved hypothetical protein	*CLR4*	FungiDB reserved gene name; Jung, et al.[14]	Deletion mutants were defective moderately in the mating ability.
CNAG _06352	1057 aa	CAP_GLY_2	197-242	Conserved hypothetical protein			
CNAG _02589	368 aa	No hit			*BDR1*	A bZIP TF for DNA damage response 1; Jung, et al.[19]	A regulator of the gamma radiation resistance of *C. neoformans*
CNAG _07593	564 aa	BZIP_ BASIC	36-50		*YAP4*	A putative Yeast AP-1-like transcription factor; Missall and Lodge[18]	Involved in induction of thioredoxin in the nitrosative condition; null mutant was sensitive to $NaNO_2$.
CNAG _07370	351 aa	BZIP	217-280	Predicted protein			
CNAG _07371	352 aa	BZIP	217-281	Predicted protein			
CNAG _07372	353 aa	BZIP	217-282	Predicted protein			

[1]Domain search was carried out by ExPasy Prosite scan analysis.
BZIP: Basic leucine-zipper (bZIP) domain profile
BZIP_BASIC: Basic leucine-zipper (bZIP) domain signature
CAP_GLY_2: CAP-Gly domain profile
[2]BZIP keyword and domain search results from *C. neoformans* H99 Database in this study

References

1 Letunic, I., Doerks, T. & Bork, P. SMART: recent updates, new developments and status in 2015. *Nucleic Acids Res.* **43**, D257-260, doi:10.1093/nar/gku949 (2015).

2 Yang, D. H. *et al.* Rewiring of signaling networks modulating thermotolerance in the human pathogen *Cryptococcus neoformans*. *Genetics* **205**, 201-219, doi:10.1534/genetics.116.190595 (2017).

3 Cheon, S. A. *et al.* Unique evolution of the UPR pathway with a novel bZIP transcription factor, Hxl1, for controlling pathogenicity of *Cryptococcus neoformans*. *PLoS Pathog.* **7**, e1002177, doi:10.1371/journal.ppat.1002177 (2011).

4 Kozubowski, L. & Heitman, J. Profiling a killer, the development of *Cryptococcus neoformans*. *FEMS Microbiol. Rev.* **36**, 78-94, doi:10.1111/j.1574-6976.2011.00286.x (2012).

5 Perfect, J. R., Ketabchi, N., Cox, G. M., Ingram, C. W. & Beiser, C. L. Karyotyping of *Cryptococcus neoformans* as an epidemiological tool. *J. Clin. Microbiol.* **31**, 3305-3309 (1993).

6 Nielsen, K. *et al.* Sexual cycle of *Cryptococcus neoformans* var. *grubii* and virulence of congenic **a** and α isolates. *Infect. Immun.* **71**, 4831-4841, doi:10.1128/iai.71.9.4831-4841.2003 (2003).

7 Kojima, K., Bahn, Y. S. & Heitman, J. Calcineurin, Mpk1 and Hog1 MAPK pathways independently control fludioxonil antifungal sensitivity in *Cryptococcus neoformans*. *Microbiology* **152**, 591-604, doi:152/3/591 [pii] 10.1099/mic.0.28571-0 (2006).

8 Bahn, Y. S., Hicks, J. K., Giles, S. S., Cox, G. M. & Heitman, J. Adenylyl cyclase-associated protein Aca1 regulates virulence and differentiation of *Cryptococcus neoformans* via the cyclic AMP-protein kinase A cascade. *Eukaryot. Cell* **3**, 1476-1491, doi:10.1128/EC.3.6.1476-1491.2004 (2004).

9 Bahn, Y. S., Kojima, K., Cox, G. M. & Heitman, J. Specialization of the HOG pathway and its impact on differentiation and virulence of *Cryptococcus neoformans*. *Mol. Biol. Cell* **16**, 2285-2300, doi:E04-11-0987 [pii]10.1091/mbc.E04-11-0987 (2005).

10 Bahn, Y. S., Geunes-Boyer, S. & Heitman, J. Ssk2 mitogen-activated protein kinase kinase kinase governs divergent patterns of the stress-activated Hog1 signaling pathway in *Cryptococcus neoformans*. *Eukaryot. Cell* **6**, 2278-2289, doi:10.1128/EC.00349-07 (2007).

11 Kim, M. S. *et al.* Comparative transcriptome analysis of the CO_2 sensing pathway via differential expression of carbonic anhydrase in *Cryptococcus neoformans*. *Genetics* **185**, 1207-1219, doi:10.1534/genetics.110.118315 (2010).

12 Kim, M. S., Kim, S. Y., Yoon, J. K., Lee, Y. W. & Bahn, Y. S. An efficient gene-disruption method in *Cryptococcus neoformans* by double-joint PCR with *NAT*-split markers. *Biochem. Biophys. Res. Commun.* **390**, 983-988, doi:10.1016/j.bbrc.2009.10.089 (2009).

13 Fraser, J. A., Subaran, R. L., Nichols, C. B. & Heitman, J. Recapitulation of the sexual cycle of the primary fungal pathogen *Cryptococcus neoformans* var. *gattii*: implications for an outbreak on Vancouver Island, Canada. *Eukaryot. Cell* **2**, 1036-1045 (2003).

14 Jung, K. W. *et al.* Systematic functional profiling of transcription factor networks in *Cryptococcus neoformans*. *Nat. Commun.* **6**, 6757, doi:10.1038/ncomms7757 (2015).

15 Paul, S., Doering, T. L. & Moye-Rowley, W. S. *Cryptococcus neoformans* Yap1 is required for normal fluconazole and oxidative stress resistance. *Fungal Genet. Biol.* **74**, 1-9, doi:10.1016/j.fgb.2014.10.015 (2015).

16 Brown, J. C. *et al.* Unraveling the biology of a fungal meningitis pathogen using chemical genetics. *Cell* **159**, 1168-1187, doi:10.1016/j.cell.2014.10.044 (2014).

17 Jung, W. H. *et al.* HapX positively and negatively regulates the transcriptional response to iron deprivation in *Cryptococcus neoformans*. *PLoS Pathog.* **6**, e1001209, doi:10.1371/journal.ppat.1001209 (2010).

18 Missall, T. A. & Lodge, J. K. Function of the thioredoxin proteins in *Cryptococcus neoformans* during stress or virulence and regulation by putative transcriptional modulators. *Mol. Microbiol.* **57**, 847-858, doi:10.1111/j.1365-2958.2005.04735.x (2005).

19 Jung, K. W. *et al.* Unraveling fungal radiation resistance regulatory networks through the genome-wide transcriptome and genetic analyses of *Cryptococcus neoformans*. *MBio* **7**, e01483-01416, doi:10.1128/mBio.01483-16 (2016).
